# Nutrient Distribution and Absorption in the Colonial Hydroid *Podocoryna carnea* Is Sequentially Diffusive and Directional

**DOI:** 10.1371/journal.pone.0136814

**Published:** 2015-09-11

**Authors:** Leo W. Buss, Christopher P. Anderson, Elena K. Perry, Evan D. Buss, Edward W. Bolton

**Affiliations:** 1 Department of Ecology and Evolutionary Biology, Yale University, New Haven, Connecticut, United States of America; 2 Smithsonian Marine Station, Fort Pierce, Florida, United States of America; 3 Department of Geology and Geophysics, Yale University, New Haven, Connecticut, United States of America; Centrum Wiskunde & Informatica (CWI) & Netherlands Institute for Systems Biology, NETHERLANDS

## Abstract

The distribution and absorption of ingested protein was characterized within a colony of *Podocoryna carnea* when a single polyp was fed. Observations were conducted at multiple spatial and temporal scales at three different stages of colony ontogeny with an artificial food item containing Texas Red conjugated albumin. Food pellets were digested and all tracer absorbed by digestive cells within the first 2–3 hours post-feeding. The preponderance of the label was located in the fed polyp and in a transport-induced diffusion pattern surrounding the fed polyp. After 6 hours post-feeding particulates re-appeared in the gastrovascular system and their absorption increased the area over which the nutrients were distributed, albeit still in a pattern that was centered on the fed polyp. At later intervals, tracer became concentrated in some stolon tips, but not in others, despite the proximity of these stolons either to the fed polyp or to adjacent stolons receiving nutrients. Distribution and absorption of nutrients is sequentially diffusive and directional.

## Introduction

Studies of the growth and form of animal colonies often come to focus on the topic of colony integration [1–3]. A principal concern is the physiological mechanisms that govern nutrient distribution amongst the tissues of a colony. Colonies can become extensive with a single organism spanning many micro-environments differing in the availability of food. Polyps capturing food must distribute nutrients to unfed tissues and, in a growing colony, to sites of growth at the colony periphery. As the colony matures, the same distribution system must direct nutrients to reproductive polyps. While these problems are shared by all colonial organisms, hydrozoans have been particularly favorable subjects for study of colony integration by virtue of the relative ease with which they can be maintained, and experimented upon, in the laboratory. It is with hydroids that we might expect the greatest clarity. Yet, our understanding of how nutrients are distributed within hydroid colonies remains incomplete, as the available data are seemingly contradictory.

The basics are not controversial. All tissues within a hydroid colony are coupled to one another by the gastrovascular system, a system of fluid-filled canals continuous with the lumen of the polyps. Upon capturing a prey item, hydroid polyps break up the food and then act as pumps driving gastrovascular fluids carrying solutes and particulates throughout the colony [4, 5]. The lumens of the stolons are lined with digestive cells and, like the gastrovascular cavity of the polyps, serve as sites of nutrient absorption.

What remains unclear is where the nutrients are ultimately delivered and absorbed as a result of gastrovascular circulation. Strehler and Crowell [6] were the first to use a nutrient tracer to detect sites of nutrient absorption. They dyed *Artemia salina* nauplii with acriflavine and noted that when fed to colonies of *Campanularia flexuosa* the dye appeared in polyps of downstream uprights within an hour. Rees et al. [7] used radiolabeled rat liver as food for *Pennaria* colonies and noted that the signal attenuated with the distance from the fed polyp. They also noted a weak, but elevated signal in the distal end of the colony at 12 hours post-feeding. This result was mirrored by Bumann and Buss [8] who fed polyps on one side of a *Podocoryna carnea* colony with labeled *Artemia* nauplii and found that the preponderance of the label remained on the fed side. However, they also showed that peripheral stolonal tips on the unfed side continued to elongate and, like Rees et al. (7), detected a weak fluorescent signal at these tips 3 days after feeding. These findings seem to simultaneously suggest that nutrient distribution favors sites adjacent to the fed polyp and sites distant from it. This conundrum motivates our attempt to further understand how nutrients are distributed and absorbed within colonies of the hydractiniid hydroid *Podocoryna carnea*.

We developed an artificial food item which can be laced with fluorescent tracers and use to track nutrient absorption. We quantified the fate of food ingested by a single polyp in an undisturbed colony. The absorption of nutrients was documented on spatial scales ranging from that of digestive cells (microns) to entire colonies (centimeters) and on temporal scales ranging from seconds to almost a week. Finally, as colonies produce different tissues at different stages of colony ontogeny, we quantified these patterns in colonies at different ontogenetic stages ranging from young colonies with few gastrozooids to sexually mature colonies bearing maturing medusae. Our results reveal previously unsuspected temporal patterns in nutrient distribution and show that colonies are capable of sequentially generating both diffusive and site-specific delivery of nutrients.

## Methods

### Animal Care

**Fig 1:**
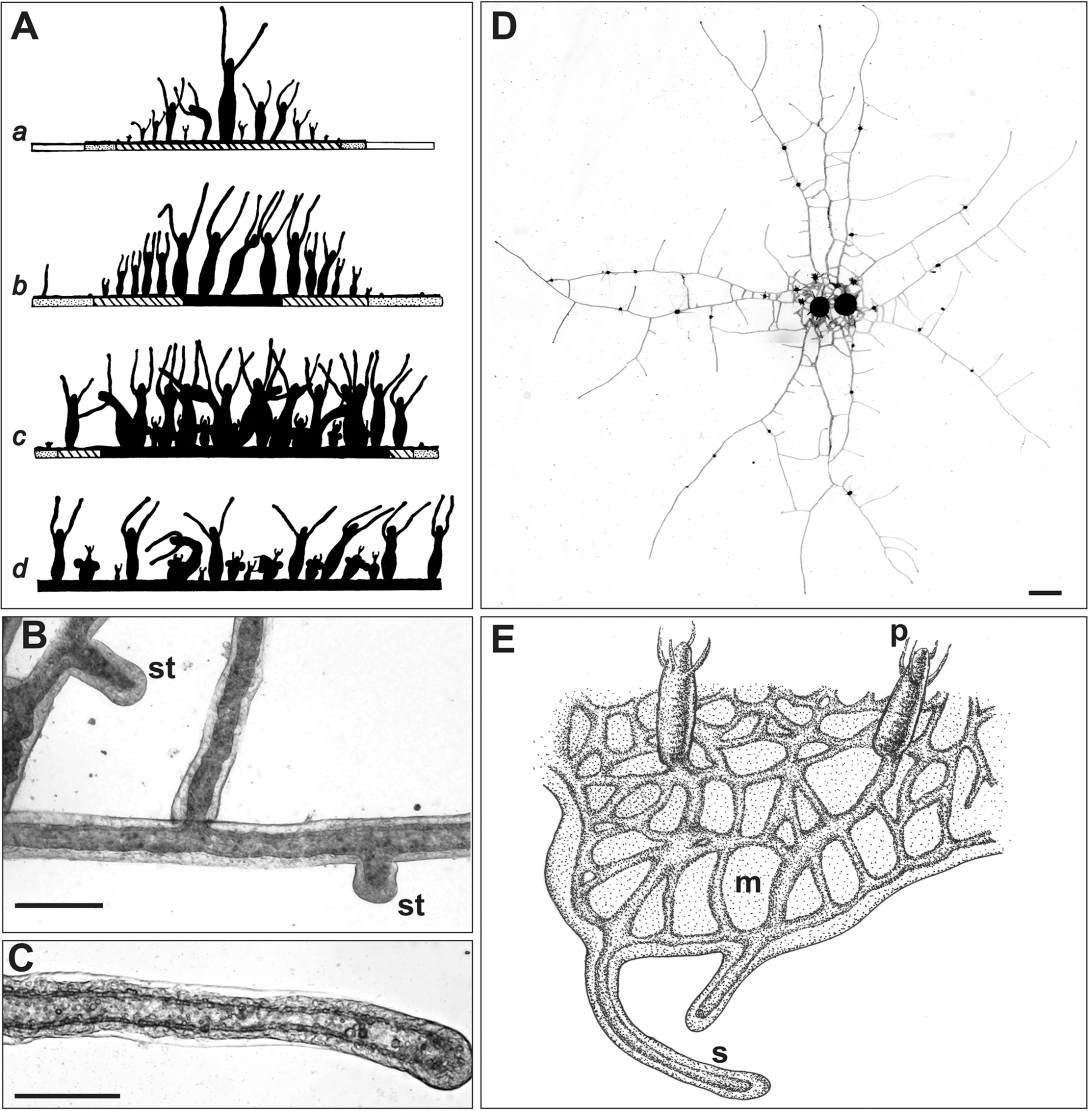
*Podocoryna carnea*. (A-D) (A) Schematic cross-section of colony viewed from the side at four different ontogenetic stages (a-d). Stippled pattern represents regions of elongating peripheral stolons. Solid pattern represents dense hydrorhizal network. Modified from Braverman and Schrandt [9]. (B) Top view of small region of hydrorhiza showing elongating stolon tips (st). Scale bar: 100 μm. (C) Longitudinal section of stolon showing lumen. Scale bar: 100 μm (D) Top view of a colony at the ontogenetic stage schematized in A subpanel a. Scale bar: 1 mm. (E) Schematic of *Hydractinia symbiolongicarpus*, showing feeding polyps (p), stolonal mat (m) and stolons emerging from mat periphery (s).

*Podocoryna carnea* is an athecate hydroid found growing as an epibiont on gastropod shells inhabited by hermit crabs and, less frequently, on shells of some living gastropods. A larva metamorphoses on a shell to produce a feeding polyp from which emanate stolons. The stolons elongate, branch and anastomose to form a hydrorhizal network, from which new feeding polyps arise (Fig 1A–1D). Specialized polyp polymorphs include tentacular and spiral zooids, which do not typically arise in laboratory culture, as well as reproductive polyps from which medusa develop. Medusae are released and complete the sexual phase of the life cycle.

This study made use of a single strain (P3) of *P*. *carnea*, collected from the intertidal of Lighthouse Point, New Haven CT in 1989. Collection was made under a permit issued by the Connecticut Department of Environmental Protection. *Podocoryna carnea* is not an endangered or protected species.

Colonies were maintained under standard conditions [10]. Briefly, colonies are grown on glass microscope slides, glass cover slips, or cover slip-bottomed culture dishes. Clonal replicates are generated by explanting a small piece of the hydrorhiza bearing 1–3 polyps and affixing it to a glass surface with a loop of quilting thread. After 2 days the colonies have attached and the thread is removed. Colonies are maintained in recirculating aquaria with daily exchanges of 25% of the seawater (31 ppt). Colonies are fed to repletion every other day with 3–4 day old *Artemia salina* nauplii. All experiments described here were performed on animals that had been fed two days earlier.

One experiment employed another athecate hydroid, *Hydractinia symbiolongicarpus* (Fig 1E). The clone utilized (LB230-16) was from a near isogenic line the pedigree for which is available elsewhere [11–13]. These colonies were propagated and maintained in the fashion outlined above for *P*. *carnea*. The differences in the anatomy of the two species germane to this investigation will be introduced at a later point.

### Nutrient Tracers

We developed an artificial food pellet that is consumed by *P*. *carnea* polyps, largely solubilizes in the gastric cavity of the fed polyp, and can be laced with fluorescent tracer molecules. The food pellets were produced from homogenized egg whites, decapsulated brine shrimp cysts, and Texas Red conjugated albumin (TRA). Texas Red was chosen as a tracer because it is a relatively pH-insensitive dye. Stock solutions of egg white were prepared by homogenizing 12 fresh egg whites in a blender at a very low speed set by a voltage controlling rheostat. Homogenized egg white was aliquoted into 1.5 ml conical tubes and stored at -20μC. Stock solutions of TRA (Invitrogen A 23017) were prepared at a concentration of 25 mg/ml in filtered seawater (FSW) and stored at 4°C.

To prepare pellets, decapsulated brine shrimp cysts (37 mg) were homogenized in 1 ml FSW using a 7 ml Kontes glass homogenizer for 20 strokes. In a 1.5 ml conical snap cap tube, 5 μl of the TRA stock solution was added to equal volumes of egg white and brine shrimp homogenate totaling 0.5 ml to form a final concentration of TRA of 0.25 mg/ml. This solution was briefly mixed on a vortex mixer, then further homogenized by hand using a tight-fitting conical plastic pestle. A cooking vessel for the pellets was prepared from a 45 mm aluminum weigh boat (Fisher 08-732-100) by creating a shallow 18 mm diameter depression in the center using the uncapped top of a 15 ml falcon tube. 0.5 ml of cooking oil was pipetted into this depression and, at room temperature, 20–30 0.1 μl droplets of albumin solution were introduced into the oil by using a 1 μl positive displacement syringe (Hamilton 86200). The dish was floated in a water bath equilibrated at 80°C and cooked for 10 minutes. Pellets were cleaned of oil by sliding them through 4–5 sequential pools of 5–10 μl of brine shrimp homogenate on a microscope slide. Thereafter the pellet was lifted with forceps and placed on the raised tentacles of the polyp intended to be fed. Pellets prepared in this fashion were of a repeatable size (average diameter: 482.7 +/- 21.8 μm, n = 9). Pellets were completely digested by polyps and no solid waste was regurgitated as occurs with animals fed crustaceans [14].

### Imaging

Colonies were imaged by using a Zeiss Lumar dissecting microscope or a Zeiss Axiovert 35 inverted compound microscope. Images were acquired with Zeiss Axiovision software. Digital imaging for events occurring on time scales of minutes or hours was performed on colonies bathed in 5 cm Petri dishes of still seawater covered with a large glass coverslip. Observations spanning days were made in a stage-mounted, flow-through culture system described earlier [15]. Briefly, the flow-through system utilizes a Warner RC-50 Ussing chamber modified to accommodate 22 x 40 mm cover slips as top and bottom to create a closed bath with an internal volume of 4 ml. Water is continuously circulated through the chamber at a rate of 5 ml/minute by means of a Harvard Apparatus PHD 2000 push/pull infusion pump fitted with 4 x 50 ml syringes. We detected no differences between the behavior of colonies in open dishes and the behavior of colonies in chambers.

Colonies were imaged in three different orientations. Unless otherwise stated, colonies were visualized in an inverted configuration, from their undersurface with the polyps facing downward into the water column. The focal plane was established in the mid-gastric region of the fed polyp. This view allowed simultaneous observation of multiple polyps and the hydrorhizal network. When the behavior of single polyps was of primary interest a side orientation was preferred, using polyps found growing on the edge of a cover slip or slide. In this case the focal plane ran through the oral-aboral axis of the polyp. A third configuration was chosen for the study of medusa-bearing colonies. These colonies were imaged with the polyps facing up with the focal plane located in the mid-gastric region of the fed polyp.

The fed polyp was much more luminous than the unfed polyps and the hydrorhiza, so two exposures were chosen to capture the relevant quantities. Unless otherwise stated images were obtained every 8 or10 seconds at both a short (250 ms-1 sec) and a long exposure (4–5 sec). The 8–10 second interval has previously been shown to capture polyp oscillations and to be adequate to prevent aliasing [14].

Colonies at three different ontogenetic stages were imaged. The first were young colonies two weeks after explanting (Fig 1Aa). These colonies were comprised of a small number of central large polyps and smaller numbers of young polyps and buds. Older colonies were comprised of numerous large central polyps, with young polyps and buds at the periphery (Fig 1Ab). Colonies at mid-ontogeny were nearer maturity, but had not begun to produce medusae. Mature late ontogeny colonies were characterized by large feeding polyps and polyps bearing medusae (Fig 1Ac and 1Ad).

The hydrorhizal network of colonies was determined after digesting the soft tissues using KOH, staining the perisarc with wheat germ agglutinin (WGA), and imaging the stained perisarc [16]. To remove tissue, colonies were relaxed for 90 seconds in 2% urethane (Sigma) and placed in dH2O for 10 minutes in a Coplin jar. Colonies were then placed horizontally in Petri dishes and digested for 10–15 minutes in 10% KOH with intermittent gentle rinsing of the surface with the KOH solution by using a Pasteur pipette until tissue digestion was complete (ca. 10–15 min). Slides were then washed 3 x 5 minutes in dH2O. WGA binds to the N-acetyl-D-glucosamine residues of chitin. Slides were incubated for 20 minutes at room temperature in 25 mg/ml WGA- AlexaFluor 555 (Invitrogen) in PBS, washed 3 x 5 minutes in PBS, and imaged in dH2O by using a Texas Red filter set (Ex 560 nm, Em 645 nm). Images were imported to Adobe Photoshop and digitized by hand using the Wacom Cintiq 24D graphics monitor.

### Analysis

Images were analyzed using Fiji (Image J). Mean luminance was quantified in user-specified regions of interest corresponding to polyps, stolons, and medusae. Polyps often bend and rotate about their base, such that their outlines span differing regions of interest (ROI) depending on the extent on their activity (S1 Fig). ROI were chosen to include the full range of area swept by a polyp over the course of the record. Because different polyps within the same colony are of different sizes and often rotate to differing degrees, absolute values of mean luminance in all but the smallest polyps within the same colony are not directly comparable. A further complication arises in the inverted orientation, as any region of interest chosen to capture polyps will include some stolons. To minimize these difficulties, mean luminance values for polyps were standardized to the maximal mean luminance of each polyp. While this standardization preserved information on the time course of nutrient absorption and the phase relations of polyp oscillations, it nonetheless prohibited quantitative comparisons between polyps in amount of nutrient absorbed. To compare polyps within a colony an ROI of fixed area was established and that area measured in the mid-gastric region of polyp at times when their orientations did not underlap stolons.

Calculations of mean luminance of stolons are not bedeviled by these difficulties, allowing direct comparisons of luminance between stolons within a colony. We assessed mean stolon luminance as a function of distance from the fed polyp and in various compass directions. For these measures, all regions overlain by polyps and all regions between stolons were excluded from the ROI, leaving only unobstructed stolons.

The three viewing orientations differ in the variability in mean luminance they displayed. The side configuration displayed the least variability, as the entire polyp is continuously in the focal plane, except for bends of short duration. The inverted (top-down) configuration was also favorable and unlike the side view, allowed observation of large regions of the colony. The top-up view, used only for studying of medusa-bearing colonies, was the most variable, by virtue of the interactions between polyps resulting from the high density of polyps in these colonies. The extent of this variability is shown in S2 Fig.

Our analysis was limited to documenting broad trends in the temporal and spatial distribution of nutrients following feeding. We are aware that our high-resolution time-series data may be fruitfully extended to treating the nutrient transport system as a dynamical system, but we do not offer such an analysis in this work.

### Equilibrial dimensions

Results of the imaging studies suggested the importance of determining the volumes of stolons and polyps in the absence of muscular activity. Time-lapse imaging of colonies exposed to urethane, menthol and MgCl, the narcotics in conventional use, showed these to disrupt activity, but not to eliminate it. Contractions of cnidarian circular muscles are mediated by non-muscle myosin II [17]. When exposed to blebbistatin, a specific inhibitor of non-muscle myosin II, neither stolonal lumens nor polyps varied in size. We quantified the equilibrial radius of the stolons, polyp buds, and medusa radial canals by exposing colonies to 0.26 mM blebbistatin. Photoinactivation was prevented by use of a red filter (gel filter 026, Lee filters #025, >580 nm) [18]. While colonies were exposed to blebbistatin, digital images were generated of the stolon lumens at 200X using a Zeiss Axiovert at 167 locations distributed though a colony, and the stolon diameters were measured. The same procedure was applied to the radial canals of 32 developing medusae. Blebbistatin-treated feeding polyps (n = 11) and polyp buds (n = 13) were visualized in side view. Polyp dimensions were converted to volumes under an assumption of radial symmetry using the methods of Dudgeon et al. (1999). A script, written in R, was used to calculate volumes and is provided (S1 Software).

### Confocal microscopy

Our imaging of nutrient distribution revealed spatial patterning in the distribution of digestive epitheliomuscular cells (hereafter digestive cells). To provide higher resolution images we obtained optical sections of fluorescent labeled tissue using confocal microscopy. Colonies were fed TRA pellets, allowed to digest for 4–6 hours and treated with blebbistatin as detailed above. Stolons were visualized live while bathed in the blebbistatin solution. Polyps and medusae were excised from the colony, immediately fixed in 4% formaldehyde, mounted on shimmed microscope slides, and visualized using a Zeiss LSM 500 laser scanning confocal microscope.

## Results

### Early Ontogeny

Study of young colonies affords the opportunity to explore the distribution of nutrients to small polyps, polyp buds and a relatively sparse vascular network connecting the explant to the colony periphery (Fig 1Aa). Raw luminance values were used to assess nutrient absorption of nutrient in small polyps and buds as they do not bend or rotate to an appreciable degree at the magnification used.

#### Polyps

**Fig 2:**
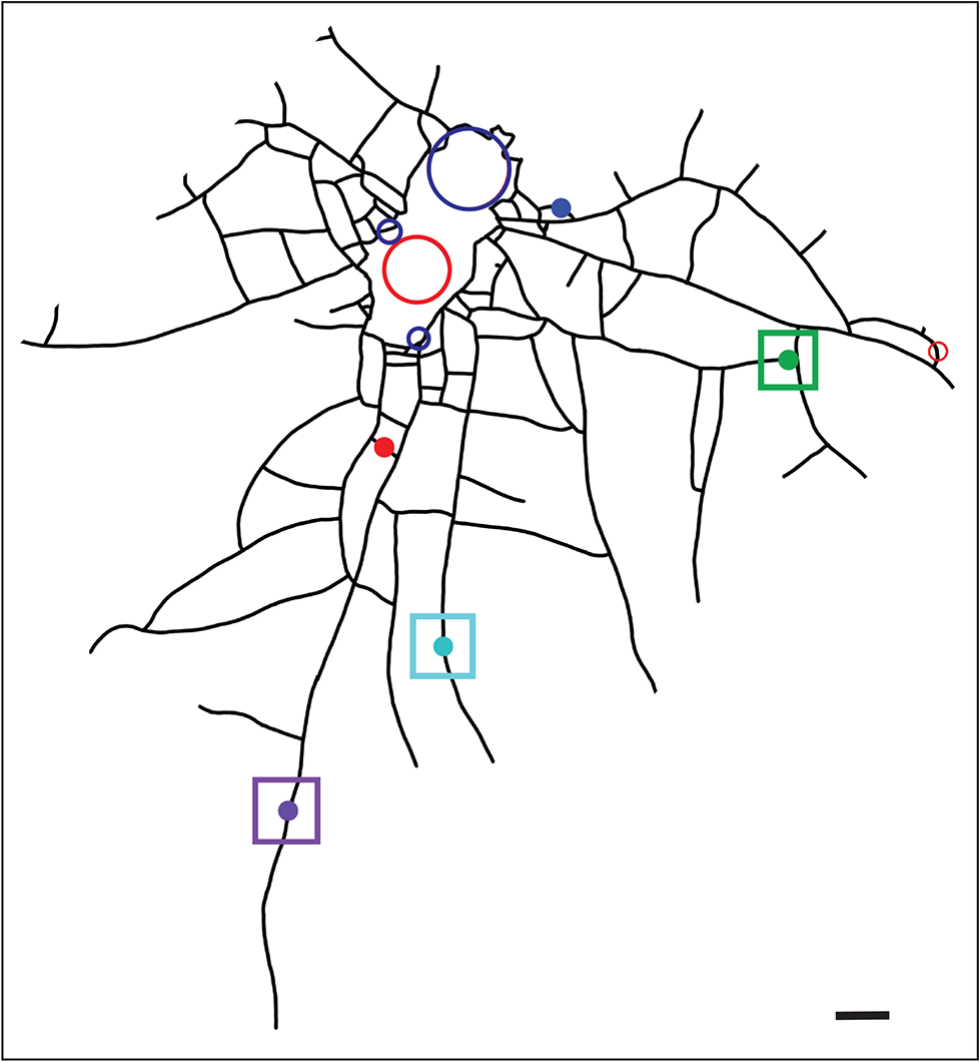
Early ontogeny colony. Closed central region represents the explant area, lines represent stolons, circles show polyp locations and their relative sizes. Fed polyp denoted as red unfilled circle, blue unfilled circles are unfed polyps. Filled circles are unfed young polyps or polyp buds. Fill colors of the closed circles are matched to the time-series shown in Figs 3 and 4. Squares are color-matched to the difference plots shown in Fig 5. Scale bar = 500 μm.

**Fig 3:**
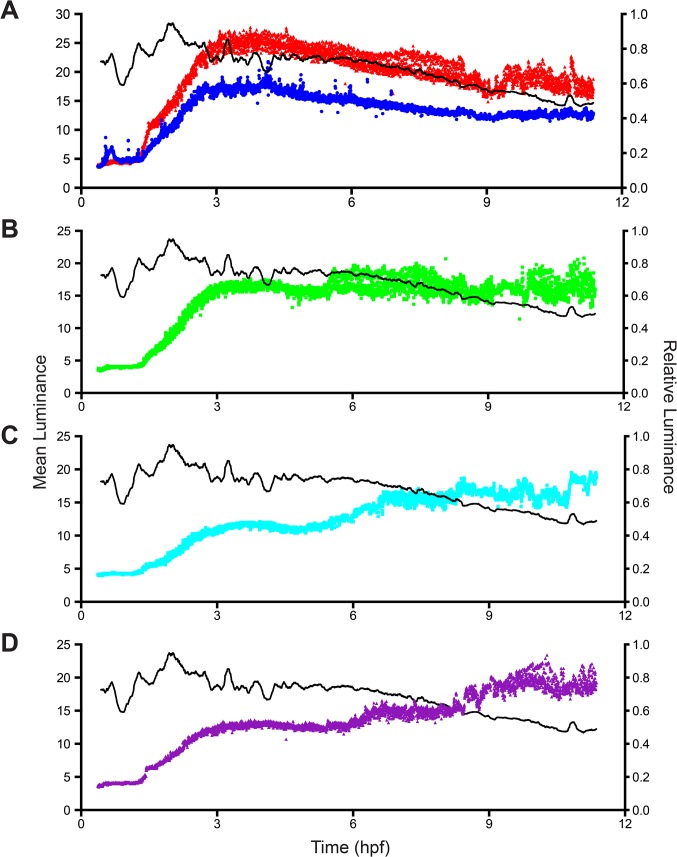
Mean luminance as a function of time for five unfed polyps. Time-series, color-coded as in Fig 1, shown for (A) two unfed polyps (red and blue in Fig 2); (B-D) one unfed polyp each (three boxed peripheral buds in Fig 3). For reference, relative luminosity of the fed polyp is shown in black. Relative luminosity was determined by standardizing mean luminosity to maximum luminosity. Time-series are color coded as in Fig 1. Sample density: 6 frames per minute, n = 3961.

The hydrorhizal network and position of polyps of the imaged colony is shown in Fig 2. The pattern of absorption in young polyps and a polyp bud is shown in Fig 3. In all unfed polyps, luminance is minimal in the 1.2 hours after feeding, thereafter increasing exponentially up to a threshold at 3.0 hours post-feeding (hpf). Observations of the fed polyp show that the pellet was completely solubilized and exported from the fed polyp by 2 hpf, consistent with the interpretation that the initial increase in luminance of the unfed polyps represents the distribution and uptake of solubilized nutrient. Additional observations of stolon lumens (see below) showed that detectable luminance disappears from the stolonal lumens shortly after the pellet is digested. The data suggest that the sigmoid relationship shown 0–6 hpf represents dissolution of the food item, rapid distribution of solubilized material and rapid and complete absorption of the labeled albumin.

After reaching the 3 hpf maximum, two of the unfed polyps lost luminance slowly over the next 8.5 hours, mirroring the decline in relative luminance in the fed polyp (Fig 3A). Both of these polyps were located near the interior of the colony (Fig 2). The remaining three polyps were located on peripheral stolons and each displayed a different pattern. One polyp retained the threshold reached at 3.0 hpf for the entire observation period (Fig 3B), but showed a pronounced increase in the amplitude of oscillations at 5.5 hpf (S3 Fig). The original sigmoid relationship found in hours 1–3 was followed by a rapid increase in luminance at approximately 6 hpf in the remaining two polyps (Fig 3C and 3D; S3 Fig). One of these displayed a third episode of increased luminance, beginning at 8.25 hpf, reaching a new threshold by 9.5 hpf (Fig 3D, S3 Fig), while the other showed a more gradual increase over the same interval. The increased luminance seen in peripheral polyps at 6–12 hpf was associated with a pronounced decrease in the relative luminance of the fed polyp, which had been largely static for the 3 hours preceding (Fig 3). The decrease in relative luminance in the fed polyp in hours 6–12 post-feeding was monotonic in contrast to episodic increases in luminance in the peripheral unfed polyps. These data indicate that the period of initial nutrient distribution and uptake (0–3 hpf), hereafter called the early distribution (ED), is followed by a later period of export of label from the fed regions of the colony towards the periphery and that during this late distribution (LD) label is either not delivered or not absorbed equally by all polyps.

#### Hydrorhiza

**Fig 4:**
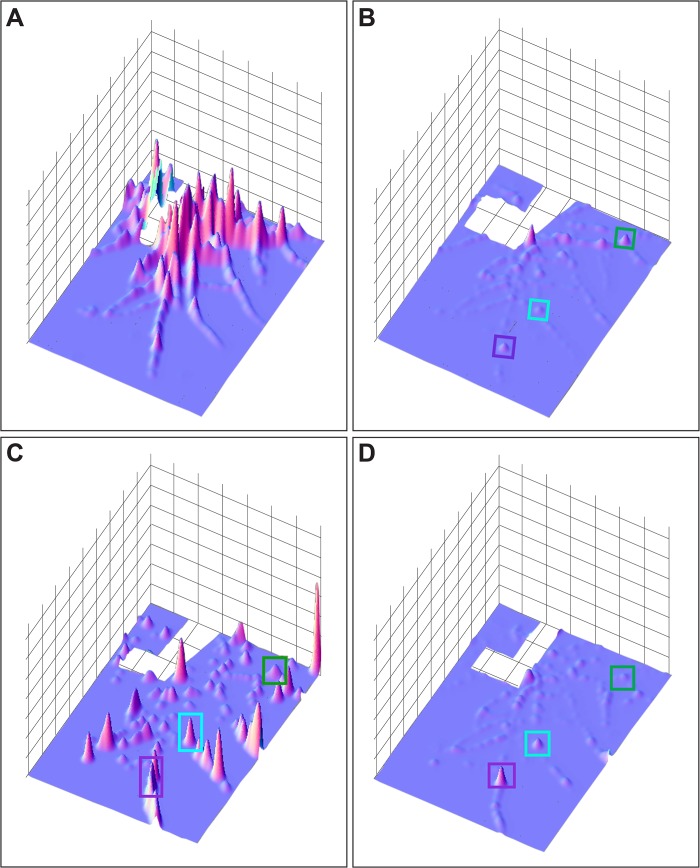
Colony wide difference plots. Z-axis is the difference in luminosity between two time points and is presented as a function of position within the colony (x-y axes). Colony orientation matches that shown in Fig 2. Differences are between (A) 0.5 and 3 hpf, (B) 3 and 6 hpf, (C) 6 and 8 hpf, and (D) 8 and 10 hpf. Boxed locations are match locations of the unfed polyps in Fig 2.

The pattern of nutrient distribution and absorption displayed by the unfed polyps was mirrored in the hydrorhizal network. The distribution of label absorption after the ED is shown as a difference plot in Fig 4A, where the x-y axis are the dimensions of the field of view and the z-axis displays difference in luminance between 0.5 and 3.0 hpf. At the end of the ED the label remains concentrated in regions surrounding the fed polyp and rapidly declines in concentration as the distance from the fed polyp increases. Note, with reference to Fig 2, that no label reaches the distal tips of the stolons. The hydrorhizal difference plot shows minimal flux during the 3–6 hpf period (Fig 4B), corresponding to the threshold observed during this same period in the unfed polyps (Fig 3).

From 6–8 hpf, during the LD, the difference plot shows enhanced signal in the hydrorhizal periphery, with label extending to distal-most regions of the stolons (Fig 4C). Comparison of the hydrorhizal network shown in Fig 2 with the LD hydrorhizal difference plots in Fig 4C shows that some stolon systems are gaining luminance while others, including some closer to the fed polyp, are not. Moreover, the lateral branches of those stolon systems that are intensely labeled are themselves not as intensely labeled. Finally, in the 8–10.5 hpf interval, the flux is minimal with label accumulating primarily in the two peripheral polyps previously identified as increasing in luminance at this period (Fig 4D). These data indicate that the two periods of distribution, ED and LD, detected in the polyps is similarly observed in the hydrorhiza. Only during the LD is transport directional.

**Fig 5:**
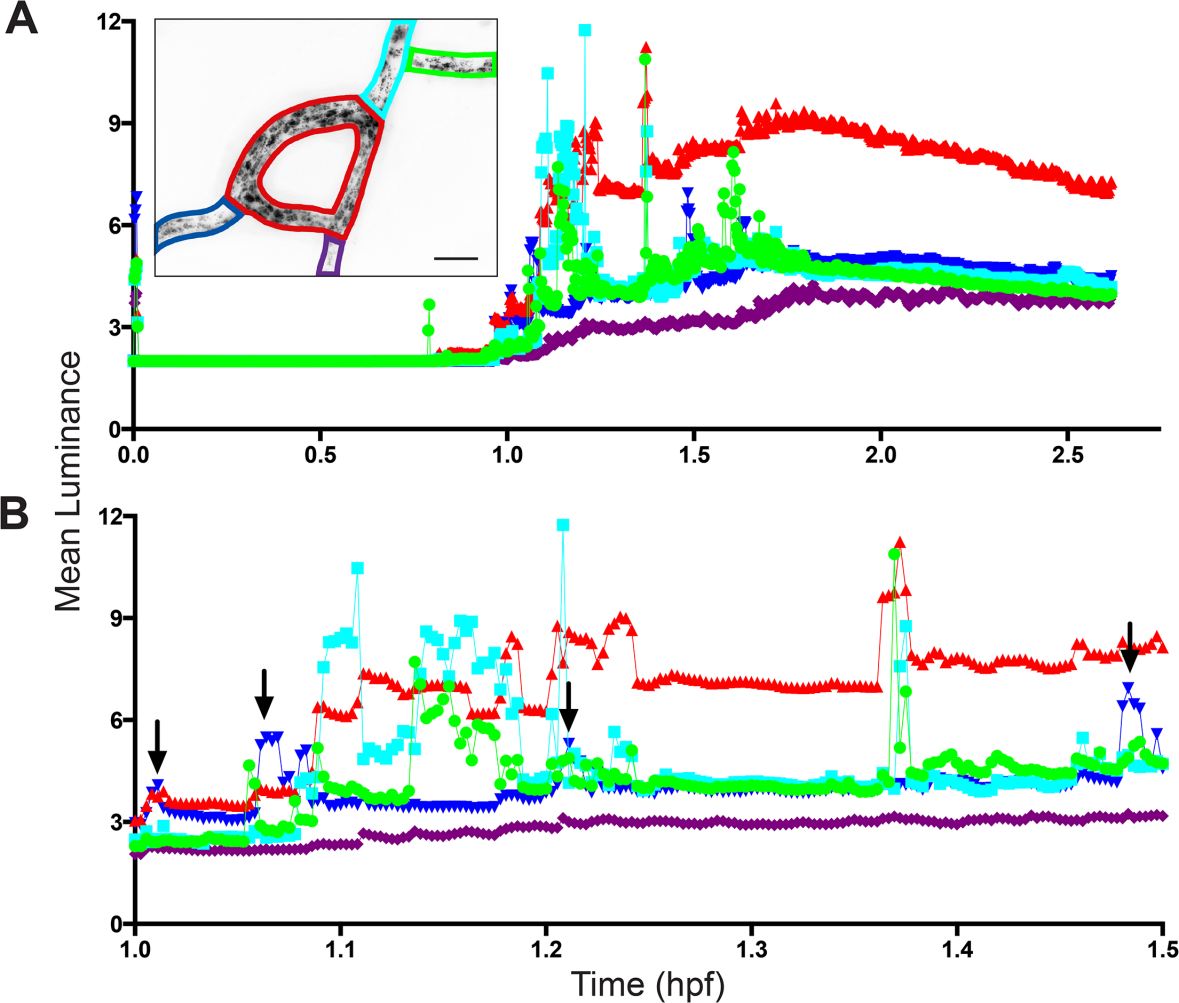
Mean luminance as a function of time for five regions of a stolon network. Inset shows network at 1:52:20 hrs:min:sec post-feeding. Network includes input segments at the upper right and two outputs at the lower left and a loop region connecting them. Inputs are in the direction of the fed polyp, outputs in the direction of the colony periphery. Scale bar = 50 μm. Colored polygons in inset are matched to symbol and line formatting in time-series. (A) Entire time series, (B) Same as (A) for a selected time window. Arrows shows spikes in output segments. Sample density: 6 frames/minute, n = 943. The images from which the data were acquired are displayed in S1 Movie.

Using a different colony, we characterized absorption of label by the stolonal gastroderm at 200X during ED. A portion of the hydrorhizal network was imaged and luminance measured for four regions of the network: two input stolon segments which first receive nutrient from the fed polyp, a closed stolonal loop that communicates with an input and two output stolonal segments (Fig 5A, inset). Label was not evident in the stolonal lumen or gastroderm in the 0–1 hpf interval, but rapidly accumulated and reached a maximum between 1.65–1.8 hpf (Fig 5A). Free label was not detected in the lumen after 1.8 hpf. Thereafter, luminance either remained constant, as in one of the output stolon segments, or decreased slightly.

The record shows several sharp spikes in luminance in the two input segments and in the loop (Fig 5A). A marked increase in the luminance of the loop segment follows each spike in the input stolon segments. Spikes sometimes appear in one of the two output segments (arrows in Fig 5B) after a variable period following the initial input spike. Inspection of S1 Movie shows that spikes coincide with unsolubilized food items that become temporarily lodged in the stolon loop. The time-delayed smaller spikes seen in the output stolon correspond to particulates exiting the loop. Several of the most prominent spikes are not followed by the particulates in the output stream; these particulates were entirely absorbed within the loop. Within the loop, digestive cells adjacent to the unsolubilized material become substantially more luminous than loop cells that have access only to solubilized nutrient (S1 Movie). This temporary clogging of the gastrovascular flow accounts for the marked greater luminance mean observed in the loop relative to both input and output stolon segments (Fig 5). The fact that cells can absorb these larger particulates shows that the digestive cells are not satiated, but that the thresholds observed in the luminance record (Fig 5) reflect elimination of nutrient from the stolonal lumen.

**Fig 6:**
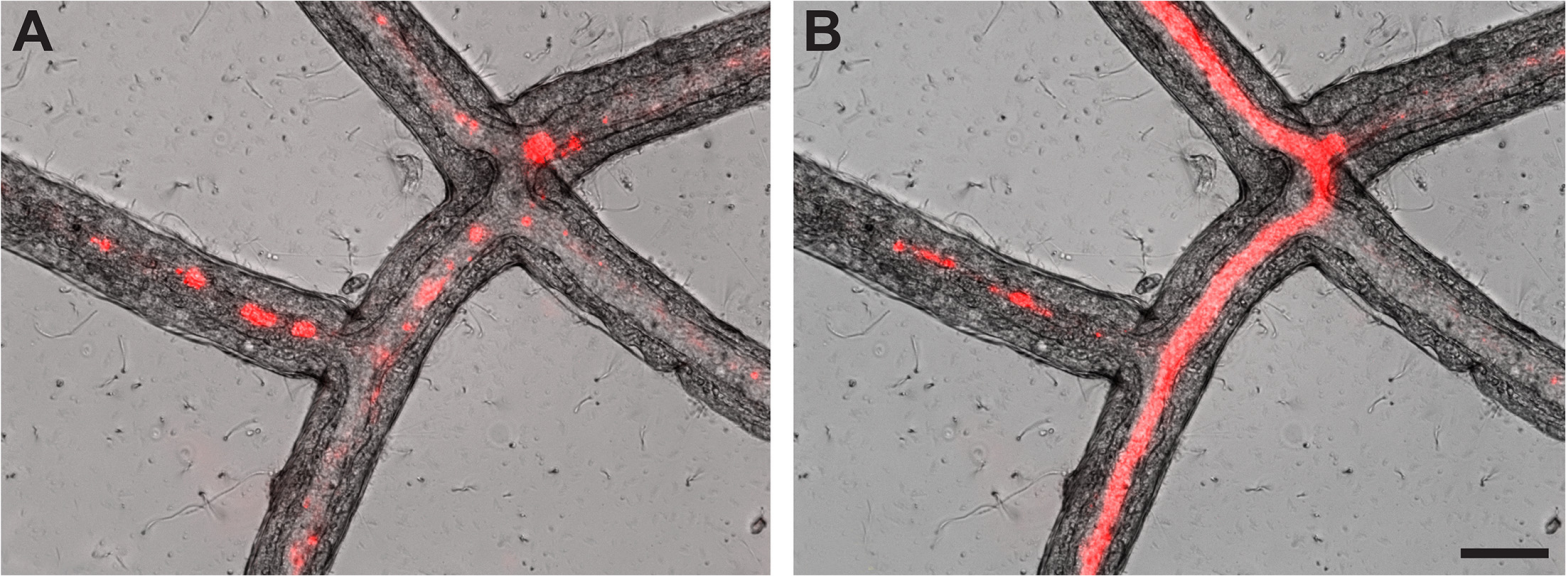
Nutrients in stolonal lumen during late distribution (A) Particulates within the hydroplasm at 12:03:37 hr:minutes:sec post-feeding. (B) Solubilized tracer at 12:08:01 hr:minutes:sec post-feeding. Scale bar = 50 μm. Additional images shown in S2 Movie.

Nutrient transport during LD was observed in stolons by filming a small region of the hydrorhiza of a different colony at 100X. S2 Movie shows that nutrients distributed during the LD can be visualized and that material appears both as particulates and solubilized material. Individual frames of this movie showing particulates and solutes appear in Fig 6.

**Fig 7:**
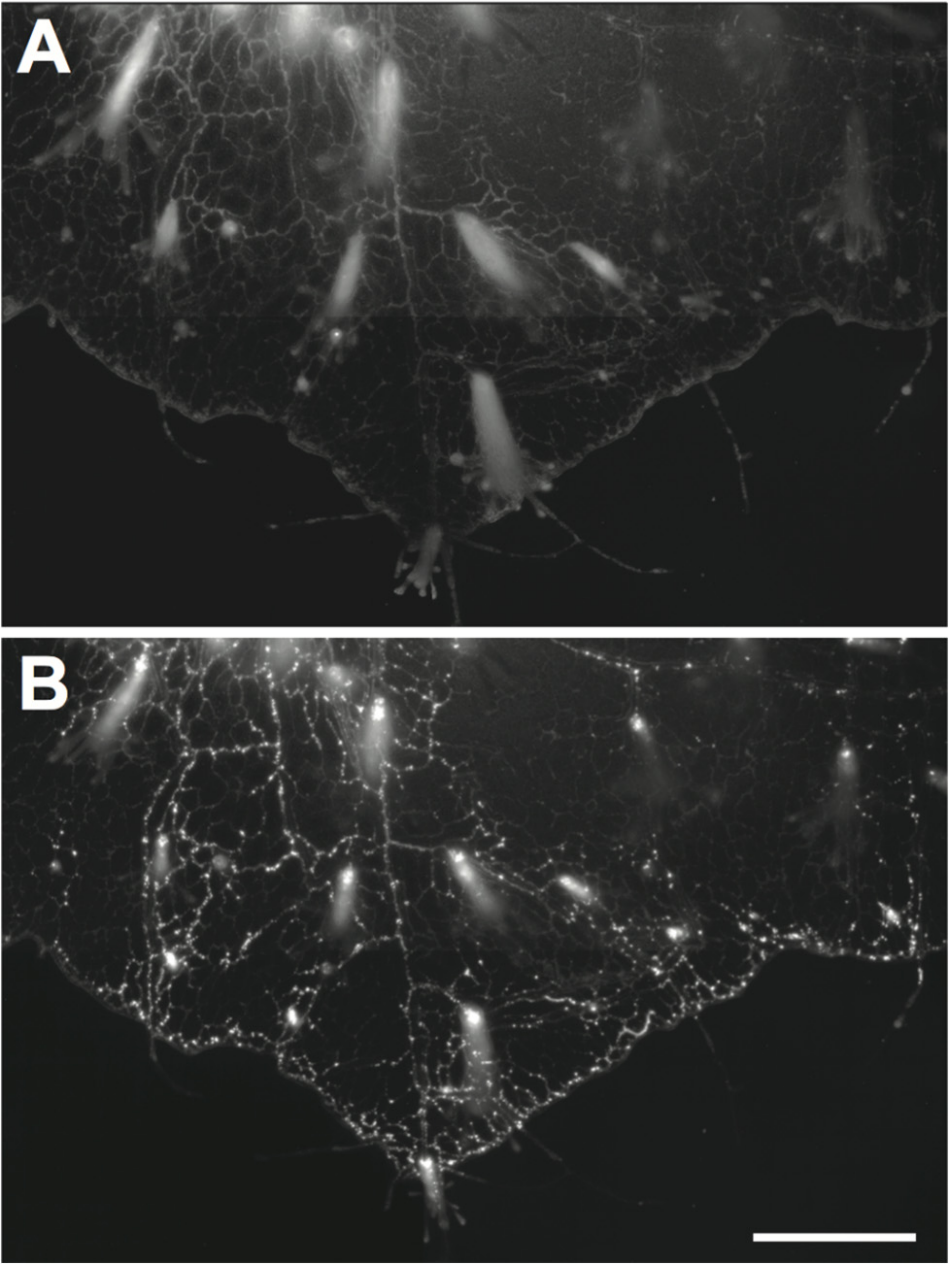
Absorption in *Hydractinia symbiolongicarpus*. (A) 5 hpf. Note even distribution in hydrorhiza diminishing in intensity toward the colony periphery. (B) 17 hpf. Note punctate pattern and wider distribution throughout colony. Frames derived from S3 Movie. Scale bar: 1mm.

Additional observations of the period of late distribution were made in a related hydractiniid hydroid, *Hydractinia symbiolongicarpus*. *Hydractinia* displays a stolonal mat (Figs 1E and 7A), an epithelial extension of the body column over the gastrodermal nexus [19]. The mat lacks perisarc, thus advantageously boosting the luminosity signal relative to that monitored in *Podocoryna*. A second advantage of *Hydractinia* for these observations is that the gastrovascular canals of the mat are substantially smaller than the stolons of *Podocoryna*, allowing a larger number of gastrodermal lumens to be observed in a single field of view. The period of early distribution is dominated by transport of solubilized material (Fig 7A), followed by a sharp transition to the period of late distribution characterized by a release from the fed polyp of large numbers of particulates (Fig 7B) that circulate widely and are only occasionally seen to solubilize (S3 Movie).

**Fig 8:**
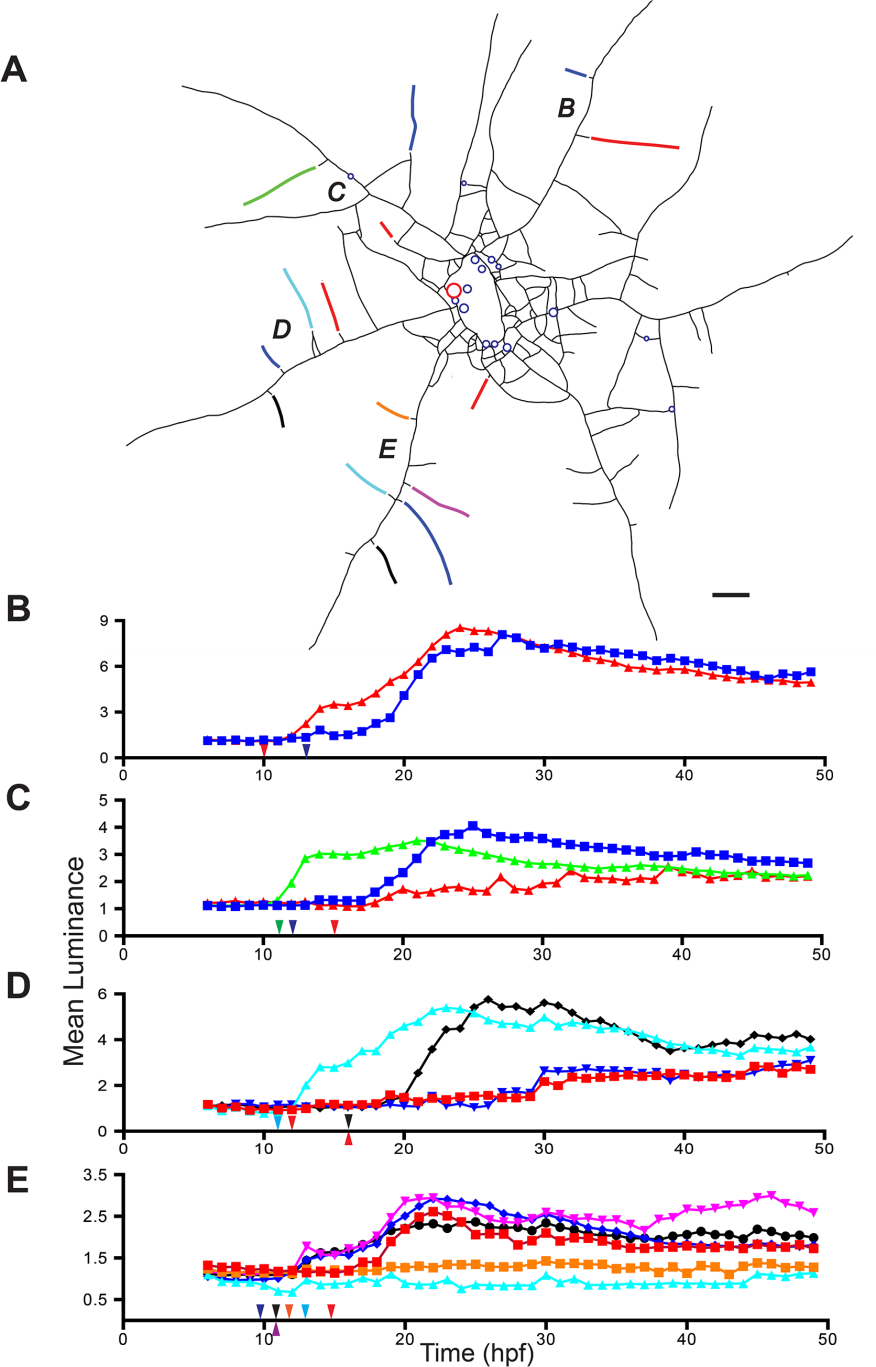
Luminance in reconnected stolons. (A) Colony outline, with stolons as line segments and polyps as open circles. Fed polyp shown in red. Stolons were severed in locations indicated by gaps. Color formatting of stolons matches time-series in (B-E). Italicized letters denote stolon systems. Scale bar = 1 mm. (B-E) Mean luminance as a function of time for stolons populating stolon systems *B-E* respectively. Arrows indicate the time at which each stolon reconnected.

The observational support for delivery of nutrients during LD shown in Figs 3 and 4, 6 and 7 and S2 and S3 Movies was supplemented by the following experiment using *Podocoryna*. Peripheral stolons were severed at 15 locations in the colony shown in Fig 8A. The colony was then fed an artificial food item bearing the fluorescent albumin tracer and the entire colony was imaged at hourly intervals from 6–49 hpf. The severed stolons were expected to reconnect at times after the ED, so that any label detected in the reconnected tips would be attributable to absorption occurring during LD. All 15 severed stolons re-connected between 10–16 hpf. Severed stolons were located on one of four major stolon systems, denoted *B-E* in Fig 8A. The respective time-series for each of their reconnected stolons are shown in Fig 8B–8E.

The timing and extent of late absorption varied both within and between stolon systems. First, two of the 15 stolons, both in stolon system *D*, did not markedly increase in mean luminance. Notably neither of these stolons was located at the extreme periphery of the system. Indeed, one of them was adjacent to two other stolons that did increase markedly in mean luminance. Note further that stolons closest to the fed polyp in several stolon systems did not display the largest mean luminance, nor were they the first stolons within a stolon system to absorb labeled protein upon reconnection. Several stolons increased in mean luminance immediately upon reconnection to the colony, while others on the same stolon system show increases only at later periods. For example, one stolon in each of the systems *B*, *C*, and *D* began to increase shortly after reconnecting in hours 10–12 post-feeding, whereas other stolons in the same systems did not increase until 19–20 hpf despite having reconnected at 12–15 hpf. Stolon system *D* bore two stolons that did not display increases until 30 hpf.

Fig 8 shows several distinct episodes of rapid increase in luminance (e.g, at 12, 20, 30 hpf). The rapidity of these increases indicates that the increased luminosity is attributable to nutrient distribution, as cell movement occurs on longer time-scales [20, 21]. The fact that in all four systems one or more stolons increased in luminance after reconnecting suggests that the polyp is distributing material colony-wide. Hydrorhizal luminosity increases neither varied predictably with the proximo-distal position of the stolon within a system (NS, Mann-Whitney U-test, n = 15, U = 112.5), nor with length of the stolon (r = 0.359) (Fig 8).

To test whether the episodic nature of the luminosity increases was attributable to episodic polyp oscillation, we imaged a fed polyp in side view for a period of 28 hpf. The polyp continuously oscillated for the entire interval, albeit with decreasing amplitude and with a pronounced reduction in period at later times (S4 Fig).

### Mid-Ontogeny

**Fig 9:**
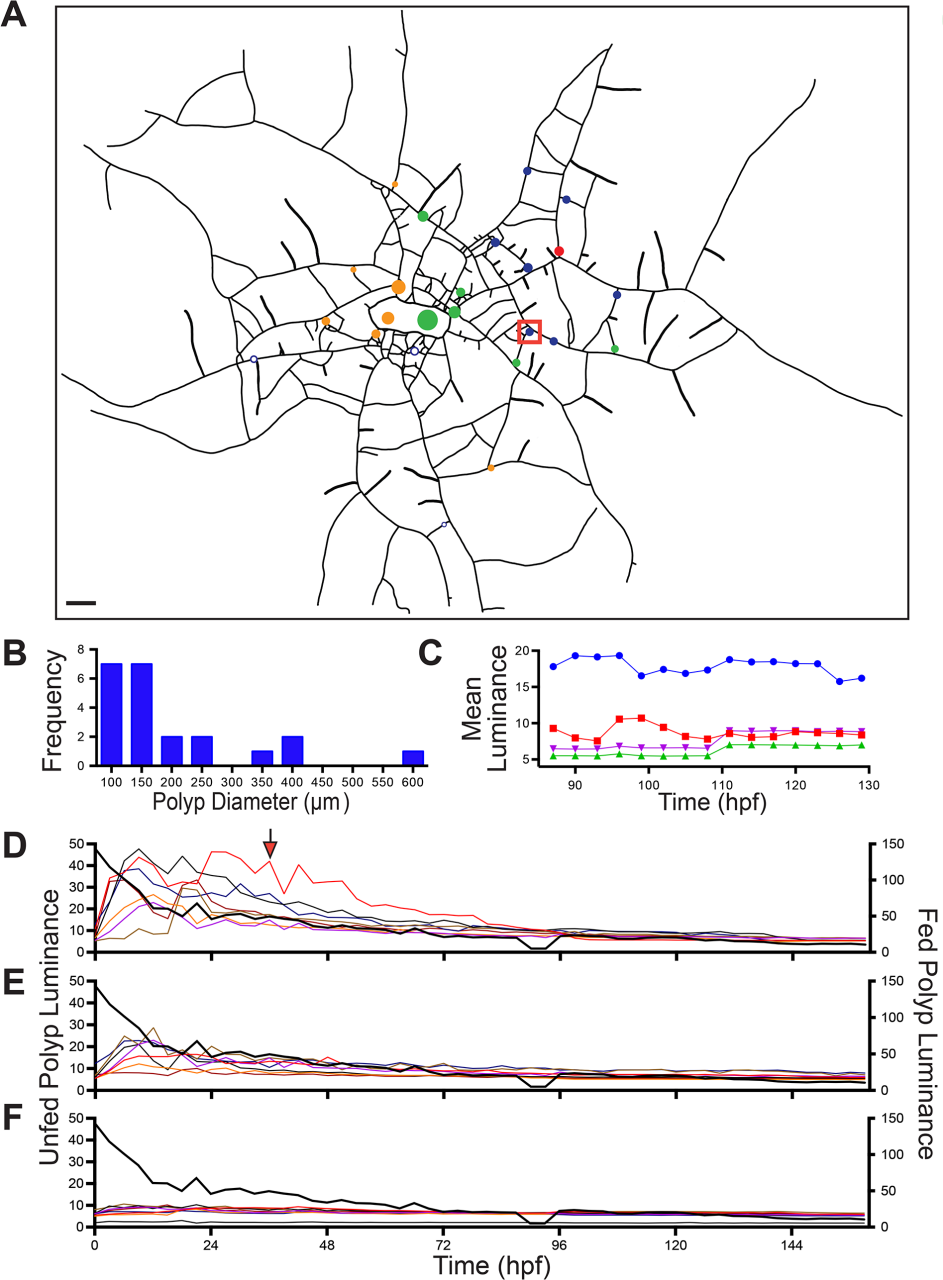
Long time course luminance record. (A) Schematic of colony. Fed polyp, red circle; unfed polyps <3 mm from fed polyp, blue; 3 > x <5 mm, green; >5mm (orange). Box corresponds to trace designated by arrow in (D). (B) Polyp size frequency distribution. Scale bar = 1 mm. (C) Luminance of distal-most 250 μm of a stolon (blue circles), and that of an identical area 1 mm from that tip (red squares), and a non-growing adjacent tip (green triangles) and a region 1 mm proximal to non-growing tip (purple inverted triangles). (D-F) Mean luminance of fed (black bold trace) and unfed polyps (D) >3, (E) 3<x<5, (F) >5 mm from fed polyp.

The preceding characterization of nutrient distribution and absorption encompassed time scales ranging from seconds up to 2 days post-feeding. A principal result was that after an initial solubilization and clearance of the food item, a period of late distribution ensued. This finding led us to extend our analysis to longer time intervals. A colony at an ontogenetic stage slightly more advanced than the colonies examined earlier was chosen (Fig 1A(b), Fig 9A and 9B). A polyp (212 μm^2^) midway between the colony center and the periphery (red circle in Fig 9A) was fed and images were acquired at 3 hour intervals for >6 days (159 hpf).

#### Polyps

Luminance of the fed and all unfed polyps are shown in Fig 9D–9F. The fed polyp luminance (black lines in Fig 9D–9F) was maximal immediately after feeding and declined markedly in the first 48 hpf, with the most rapid decay occurring in the first 24 hpf. After the second day, decay in luminance was gradual and was comparable to that displayed by unfed polyps. The luminance of unfed polyps varied as a function of the distance from the fed polyp (Fig 9D–9F). Polyps within 3 mm of the fed polyps were the most luminous (Fig 9D), polyps >3 and <5 mm were less luminous (Fig 9E) and those >5 mm distant showed no increases in luminance throughout the record (Fig 9F). Increases in luminance in the nearby unfed polyps paralleled the decline in luminance of the fed polyp, with unfed polyp luminance reaching a maximum within the first 24 hpf and declining to a common baseline luminance within 48–72 hpf. One small (120 μm^2^) unfed polyp displayed a markedly different temporal pattern, showing a maximal luminance within the 24–48 hpf and a decay to baseline values only after 84 hpf (box in Fig 9A, arrow in Fig 9D).

#### Hydrorhiza

The pattern of absorption within the hydrorhiza is shown in S4 Movie. Luminance was observed in a radial pattern surrounding the fed polyp in the first 24 hpf. By 48 hpf, hydrorhizal luminance was found at a substantially greater distance from the fed polyp and was localized toward the colony periphery. Note that maximal luminance in all but one of the unfed polyps was reached in first 24 hpf and thereafter declined rapidly (Fig 9). The coincidence of the decline in luminance from the polyps with increased luminance of the hydrorhiza suggests preferential late transport to the colony periphery at longer time scales. At 3–6 days post-feeding, luminance was diminished throughout, as the tracer was digested.

**Fig 10:**
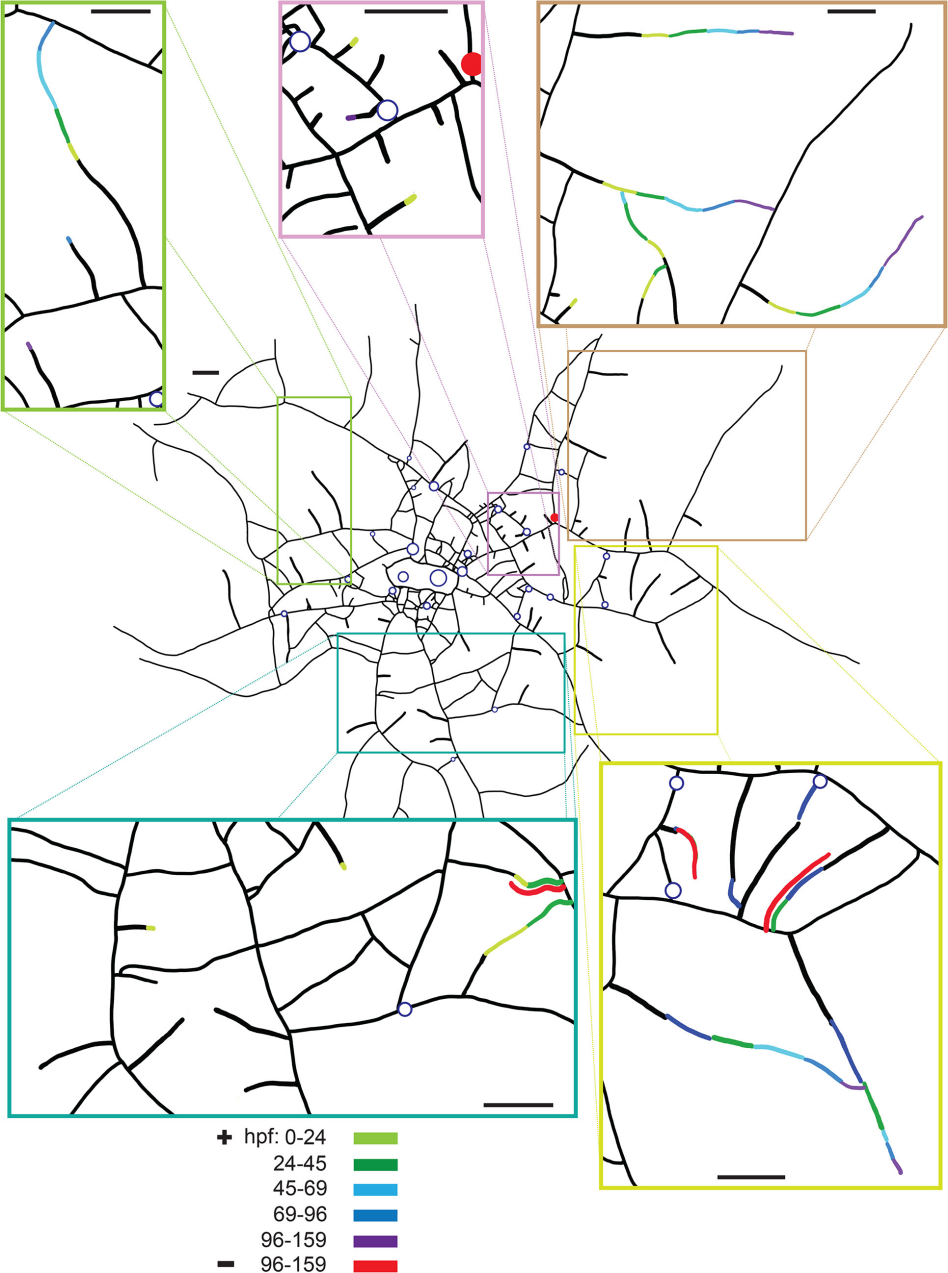
Stolon growth (+) and retraction (-) in selected regions at five time intervals (hpf). Fed polyp filled red. Unfed polyps blue open circles. Where retractions (red) overlapped locations of prior growth (any other color), the retractions are drawn parallel to the regions of growth. Scale bars = 1 mm.

The long duration of these observations permitted us to document stolon growth (Fig 10). In the first two days after feeding, most stolons were not observed to grow and those that did grow were distributed throughout the colony. From two days post-feeding until the termination of observations after 6+ days, only a few isolated stolons were found to grow, but these active stolons increased in length by several mm (Fig 10). In the fifth and sixth day post-feeding some stolon tips retracted, although the extent of the retraction was small relative to the elongation observed in growing tips. While most of the actively growing stolons were found in regions of the colony periphery nearest the fed polyp, one stolon on the opposite side of the colony center was observed to grow several mm. Moreover, several stolon tips immediately adjacent to the fed polyp displayed no growth.

Stolons that were growing 3–6 days post feeding were observed to continue to receive labeled albumin at these times. Growing stolon tips were characterized by enhanced luminance relative not only to non-growing tips, but also to more proximal regions of the same tip (Fig 9C, S5 Movie). These observations are reminiscent of the experiment where stolons were severed to detect absorption of proteins during the period of late distribution (Fig 8), where nutrient absorption was similarly found to occur in only a subset of the stolons.

### Late Ontogeny

Study of immature colonies afforded the opportunity to explore the distribution of nutrients to small polyps, polyp buds and a relatively sparse vascular network connecting the explant to the colony periphery. Imaging of colonies later in development (Fig 1Ac–1Ad) allows study of polyps over a range of sizes and, at maturity, investigation of the behavior of medusa-bearing reproductive polyps.

#### Polyps

**Fig 11:**
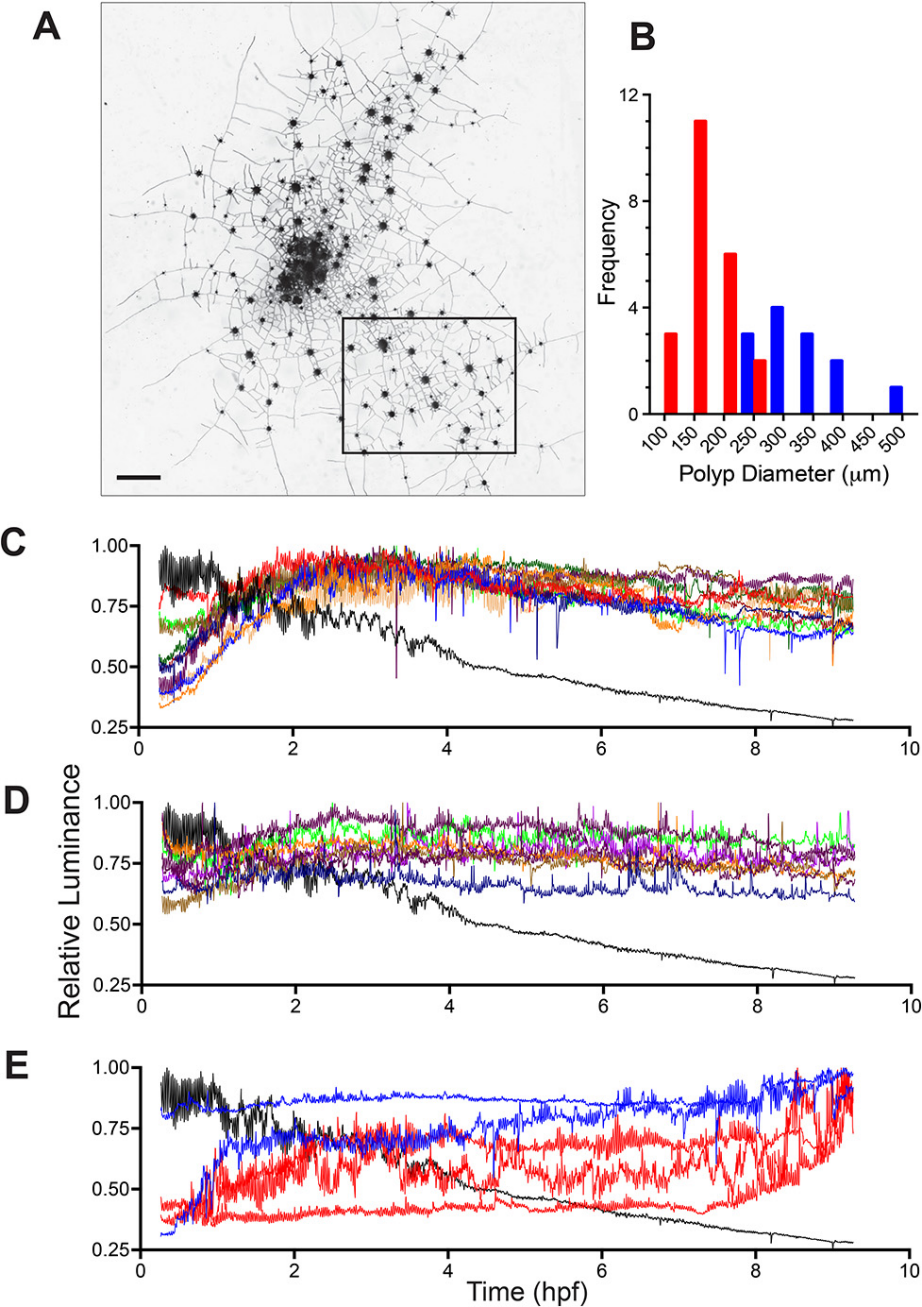
Late ontogeny colony. (A) Photomicrograph of entire colony with the region imaged enclosed by the rectangle. This region is shown at higher magnification in Fig 12A. Scale bar = 2 mm. (B) Size frequency distribution of polyps. Color formatting matched to time-series in (E). (C) Luminance standardized to maximal luminance (i.e., relative luminance) as a function of time for 10 different large unfed polyps and the single fed polyp. Fed polyp in black. (D) Relative luminance as a function of time for 8 small unfed polyps and the single fed polyp. Fed polyp in black. (E) Relative luminance of two additional large (blue) and 3 additional small (red) unfed polyps. Fed polyp shown in black. Sampling interval: 6 frames/minute, n = 3241.

A colony at the ontogenetic stage just prior to the formation of medusae is shown in Fig 11A. Note that only a portion of such colonies can be imaged at high time-resolution, as the polyp oscillations occur on a time scale (ca. 90 seconds) that requires that a field of view remains fixed if the aliasing of the oscillation is to be avoided. Colonies at this stage show a range of sizes (Figs 1Ac and 11B). The majority of the smaller polyps will mature into reproductive polyps, but at this stage they remain competent to feed.

The relative luminance of 10 large unfed polyps and 8 small unfed polyps are shown in Fig 11C and 11D, respectively. The relative luminance of the large unfed polyps increases rapidly in the first two hours post-feeding and thereafter changes little or slightly declines. Small unfed polyps display a similar pattern, differing only in that they reach a threshold approximately an hour later than the fed polyps. The food pellet was largely solubilized by the fed polyp by 2.25 hours. Note that the luminosity of the fed polyp (black trace in Fig 11) continues to decline even after the unfed polyps have reached their maximal luminance.

Five polyps displayed absorption of labelled protein during LD, with one polyp increasing in relative luminance from 4 hpf and the other four increasing in relative luminance at 7.5–8.0 hours post-feeding (Fig 11E). During this same interval, the fed polyp displayed no change from the monotonic decay begun at 4 hpf. All five of these polyps were located in the northwest quadrant of the imaged region, toward the center of the colony. In contrast all polyps that failed to show absorption of nutrients during LD (Fig 11C and 11D) were located in the other three quadrants, all closer to the colony periphery.

The patterns in relative luminance seen in colonies late in ontogeny are in broad accord with those displayed by the colonies at early stages of ontogeny (Figs 3 and 9). Both stages are characterized by rapid increases in luminance as the food pellet decays, reaching a threshold that is either maintained or decays slightly, with one or more episodes of later transport to some, but not all polyps distant from the fed polyp.

**Fig 12:**
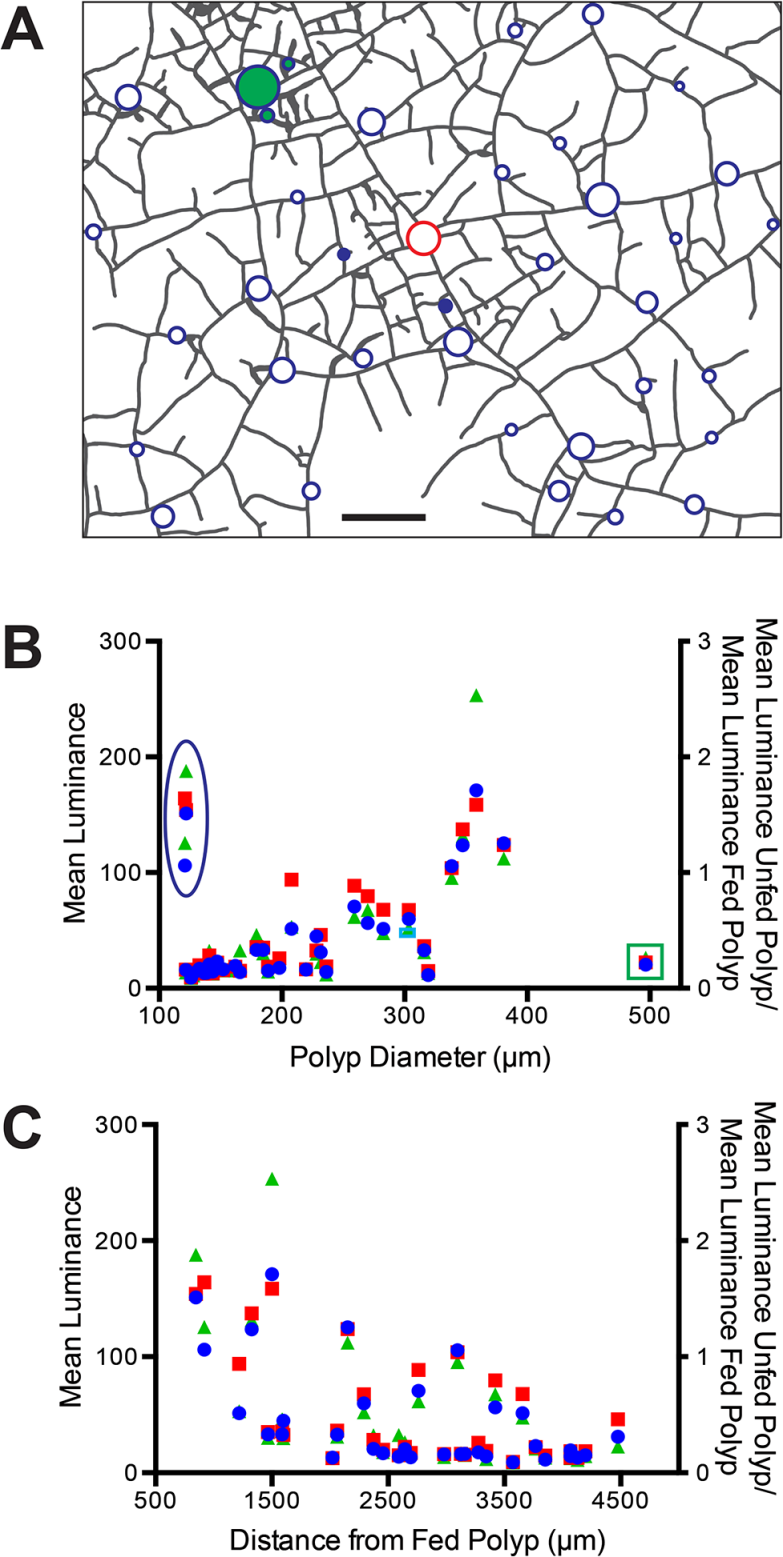
Polyp size and position. (A) Colony outline. Fed polyp indicated as red unfilled circle, unfed polyps as blue unfilled circles. Filled circles are unfed polyps, with the fill color-coded to match highlighted data points in B. Scale bar = 1 mm. (B) Mean luminance of unfed polyps as a function of polyp size at three time points: 1.5 hpf, blue circles; 4 hpf, red squares; 9 hpf green triangles. Additional scale shows mean luminance of unfed polyp standardized to the mean luminance of the fed polyp. Data points enclosed within the ellipse are derived from the polyps indicated as filled blue circles in A, those enclosed within the box are derived from the polyp shown as a filled green circle in A. (C) Mean luminance of unfed polyps as a function of the distance from the fed polyp. Axes and formatting of data points as in B.

The broad size range of the polyps in colonies at this ontogenetic stage (Fig 11B) led us to investigate the relationship between polyp size and luminosity (Fig 12). Mean luminosity for a constant region of interest was determined for all polyps at three time points: 1.5, 4, 9 hpf. A weak positive relationship of luminosity with polyp size was detected, with several outliers (r = 0.331 at 4 hpf; r = 0.333 at 9 hpf). Polyps displaying high luminosity at small polyp sizes were located near the fed polyp (blue filled circles in Fig 12A and ellipse in Fig 12B, B respectively). Conversely, a large polyp displaying low luminosity was observed far from the fed polyp (green filled circle in Fig 12A and box in Fig 12B respectively). Examination of these outliers prompted examination of the mean luminosity as a function of the distance from the fed polyp (Fig 12C). A pronounced decay in luminosity with distance was detected (r = -0.594 at 4 hpf; r = -0.586 at 9 hpf). Data from differing time points were comparable as expected from the thresholds evident in Fig 11. The only notable exception was a polyp in the northwest quadrant previously detected as a polyp absorbing nutrient from 4 hpf. The relationships between luminosity and polyp size and with distance are also shown standardized to the mean luminance of the fed polyp (right y-axis in Fig 12B and 12C). Note that the majority of the unfed polyps have values <1, indicating that the fed polyp retains the majority of the fluorescent albumin and that only large and/or nearby unfed polyps exceed the fed polyp in luminance after 9 hpf.

**Fig 13:**
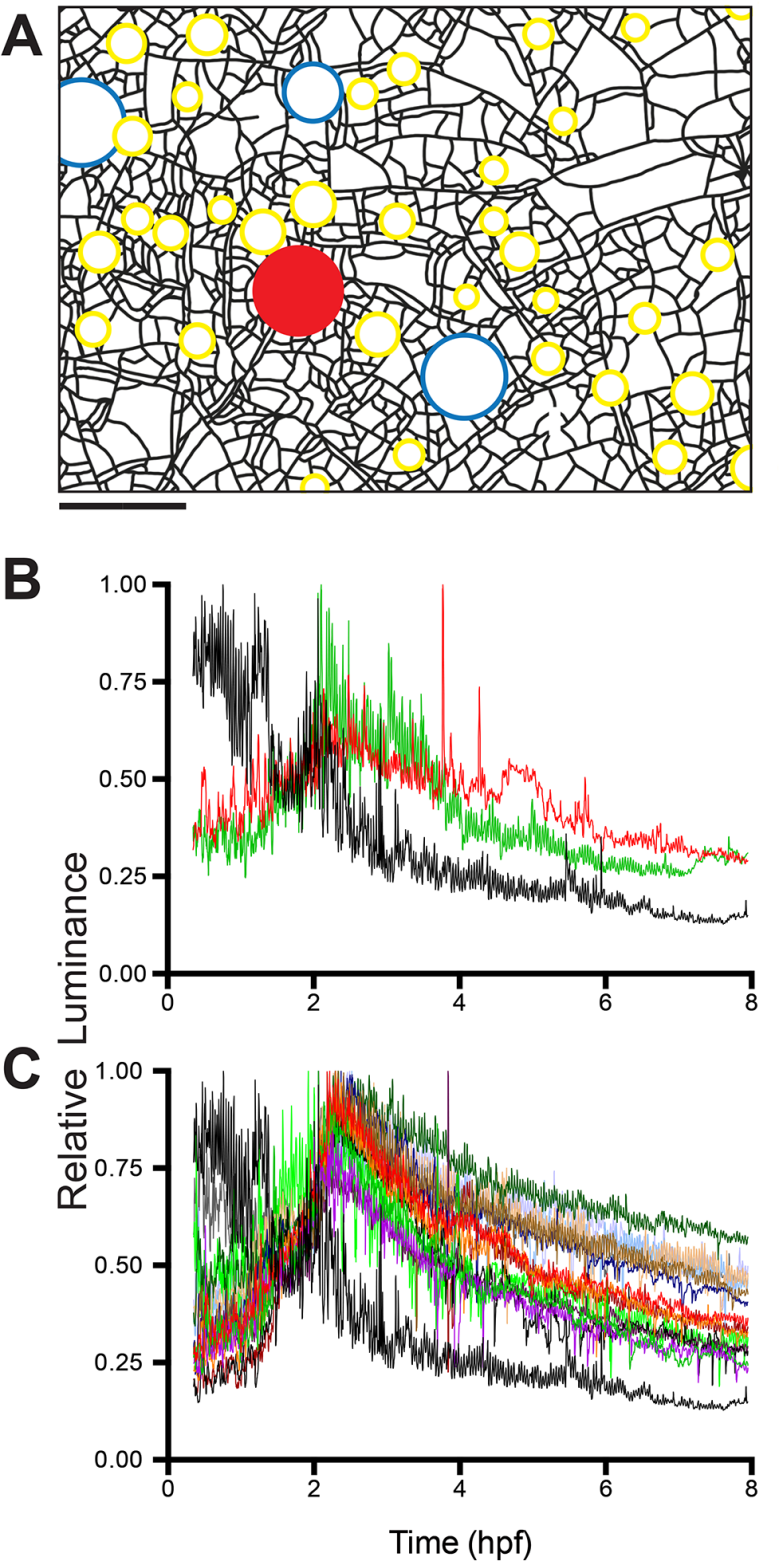
Reproductive colony. (A) Schematic of field of view of a medusae-bearing colony. Fed polyp in red, unfed polyps outlined in blue, medusae-bearing polyps outline in yellow. Scale bar = 1 mm. (B) Relative luminance time-series for the fed polyp (black) and two unfed polyps (green, red). (C) Relative luminance for the 15 medusae-bearing polyps. Fed polyp shown in black. Sampling interval for (B,C): 6 frames/minute, n = 2737.

**Fig 14:**
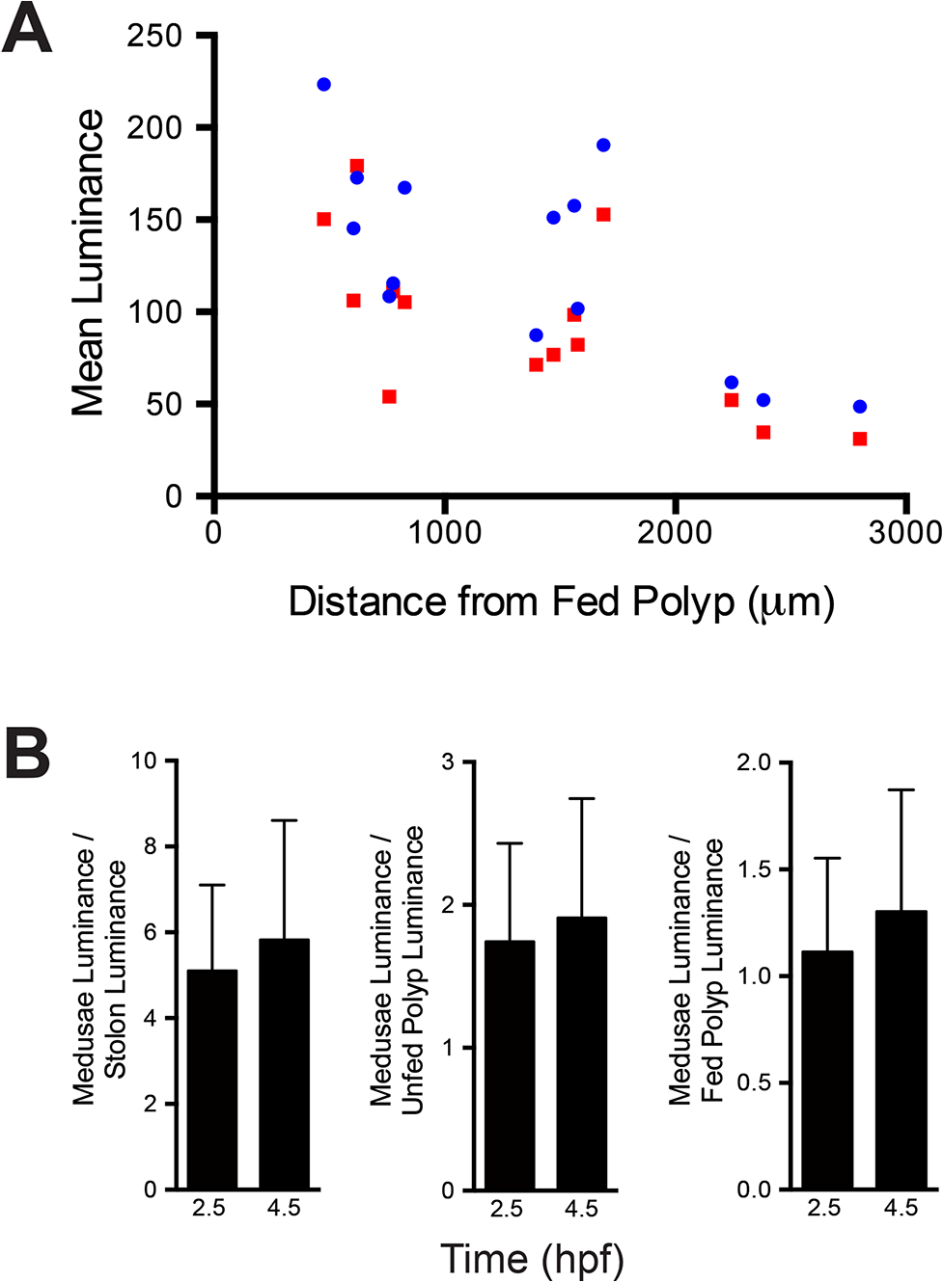
Medusae luminance. (A) Mean luminance of medusae as a function of their distance from the fed polyp at 2.5 (blue) and 4.5 (red) hpf. (B) Mean luminance of medusae standardized to mean luminance of adjacent stolons, unfed polyps, and fed polyps at 2.5 and 4.5 hpf. Errors bars show one standard deviation.

Colony ontogeny culminates with a dense hydrorhizal network which bears large feeding polyps and reproductive polyps bearing medusae (Fig 1A(d)). Such a colony is shown in Fig 13A and the relative luminance of the unfed polyps and reproductive polyps shown in Fig 13B and 13C, respectively, and in S6 Movie. Unfed polyps increase in relative luminance to a maximum at 2.5 hpf, but thereafter display a pronounced decline in relative luminance (Fig 13B). This time-course differs from colonies at earlier stages of ontogeny in which decays in relative luminance were either modest or undetected within 8–10 hpf (Figs 3 and 11). A slight increase in relative luminance, reflecting absorption of label during late distribution, was detected in one of the two unfed polyps commencing at 7.2 hpf. The medusa record is similar to that shown by unfed polyps: a maximum is reached at 2.5 hpf followed by a pronounced decline in relative luminance (Fig 13C). No increases in relative luminance during the interval of late distribution were detected in reproductive polyps. Note, however, that the fed polyp, as in previously discussed colonies (Figs 3 and 11), declines in luminance markedly from 2–8 hpf, indicating that nutrient distribution is occurring. The mean luminance of medusae varied with the distance between the reproductive polyp and the fed polyp (r = -0.704 at 2.5 hpf; r = -0.670 at 4 hpf) (Fig 14A).

The rapid decline of label in colonies bearing medusae likely reflects the substantially greater absorptive surface area in reproductive polyps bearing medusa buds than in other tissue compartments. Fig 14B shows the medusa luminance standardized to adjacent stolons, unfed polyps, and the fed polyp respectively at 2.5 and 4.5 hpf. Medusae are 5 times as luminous as adjacent stolons (p<0.001, Student’s t-test for difference from 1:1 ratio, n = 12, t = 7.67 at 2.5 hpf, t = 5.95 at 4.5 hpf), >1.75 times more luminous than unfed polyps (p<0.001, Student’s t-test, t = 5.18, n = 22, at 2.5 hpf, t = 5.34, n = 23, at 4.5 hpf) and are as luminous or slightly more luminous than the fed polyp (Student’s t-test, NS, t = 1.24, p = 0.22, n = 22, at 2.5 hpf, t = 2.61, n = 23,p<0.05 at 4.5 hpf).

#### Hydrorhiza

**Fig 15:**
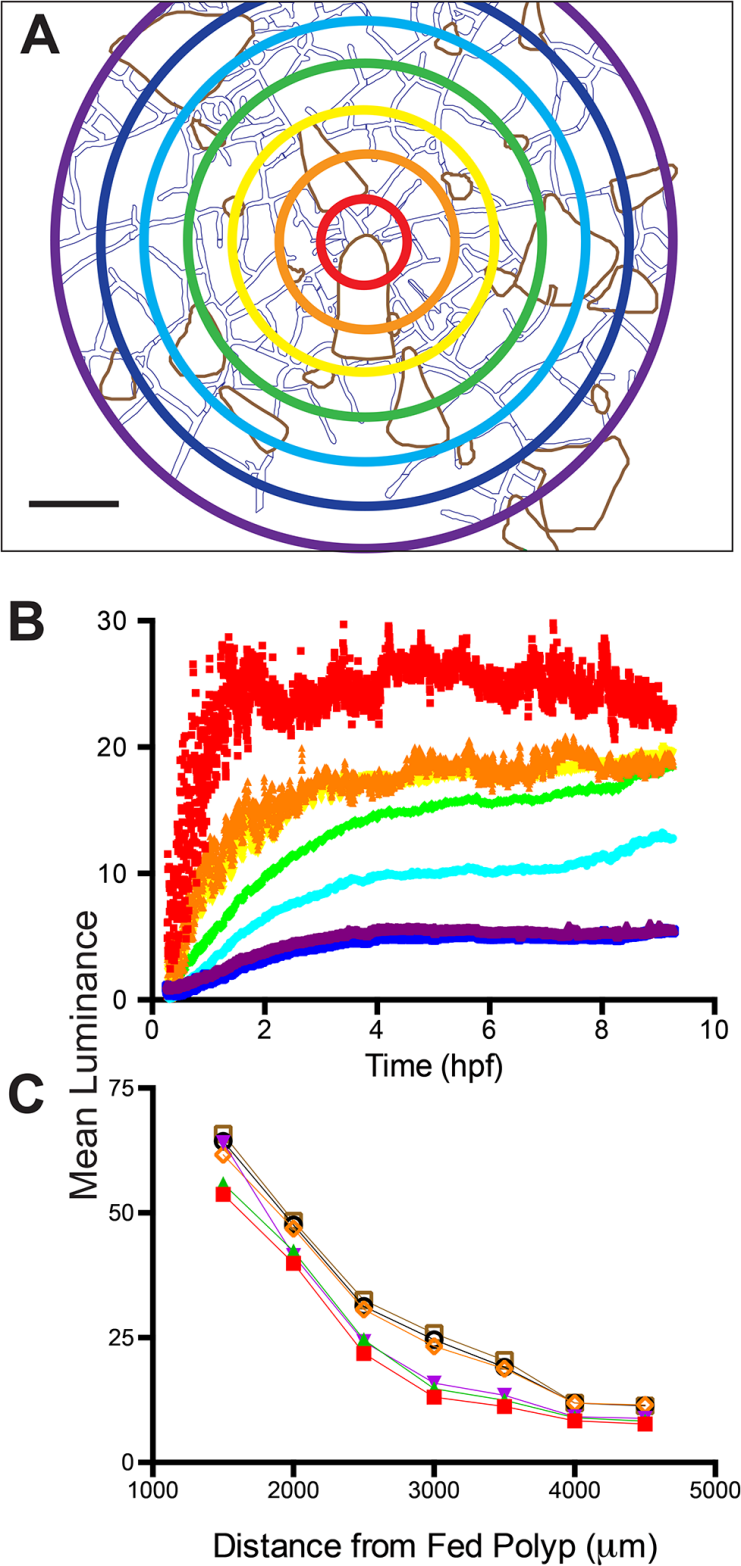
Hydrorhizal luminance in late ontogeny. (A) Superimposed on the colony schematic are seven concentric circles that increase in radius in steps of 0.5 mm. Measurements were made in the annuli between circles by outlining the stolons and calculating their mean luminance. Irregular areas defined in brown were not measured. These regions correspond to locations where polyps obscured underlying stolons in part or all of the record. Color of circles matched to time-series shown in (B). Scale bar = 1 mm. (B) Time-series of mean luminance for each annulus. Sampling interval: 6 frames/minute, n = 3241. (C) Mean luminance as a function of distance from fed polyp at 0.85 (closed red), 1.125 (closed green), 1.4 (closed blue), 4 (open orange), 6 (open black), 8 (open brown) hpf.

Colonies in late ontogeny display a greater density of stolons and higher connectivity than younger colonies (compare Figs 2,9A to Figs 11A, 12A). To quantify the distribution and absorption of the nutrient tracer in older colonies we measured mean stolonal luminance in annuli about the fed polyp (Fig 15A). Mean luminance was strongly dependent upon the distance from the fed polyp (Fig 15B and 15C). Nearby regions reached a threshold at 2.25 hpf (red and orange in Fig 15A and 15B), mirroring the time course observed in unfed polyps (Fig 11). More proximal regions continued to increase in luminance more gradually, as expected for absorption occurring during the interval of late distribution. The outermost region at the periphery increased little after 4 hpf, suggesting a limit to the spatial extent to which materials can be distributed before they are fully absorbed (Fig 15B). Mean luminance in the hydrorhiza behaves much like a diffusion process (Fig 15C), albeit on a network, with the periods of early and late distributions characterized by different diffusion coefficients.

**Fig 16:**
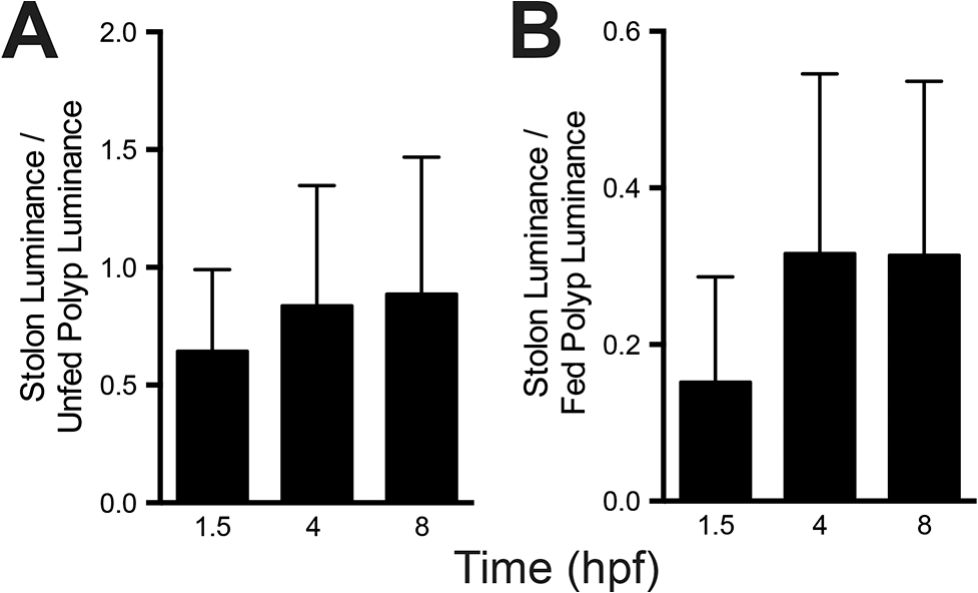
Luminance of hydrorhiza relative to polyps in late ontogeny. (A) Mean luminance of stolons standardized to the mean luminance of an adjacent unfed polyp (n = 34) at 1.5, 4 and 8 hpf. Errors bars show one standard deviation. (B) Mean luminance of stolons to mean luminance of the fed polyp at 1.5, 4 and 8 hpf (n = 34). Errors bars show one standard deviation.

Recall that the field of observation in a late ontogeny colony was embedded within a larger colony and that the polyps that received label during the period of late distribution were located in the region of the field of view closest to the center of the colony (Fig 11). We explored whether a similar pattern occurred in the hydrorhizal luminance record. The field of view was divided into wedges centered on the fed polyp with an internal angle of 45° (S5 Fig). The stolon record mirrored the results seen in the polyp record (Fig 11). Stolons in the east, south, and west regions of the colonies reached a threshold in luminance, with no suggestion of additional absorption during the LD period. In contrast, stolons in the direction of the colony center, those in the northwest, north and northeast, displayed a signature of absorption during the LD interval. Mean luminance of the hydrorhiza was roughly half that of adjacent unfed polyps at early times (p<0.001, Student t-test for 1:1 ratio, n = 33, t = 5.99 at 1.5 hpf) and roughly equal to unfed polyps at late times (p = 0.072, Student t-test for 1:1 ratio, n = 33, t = 1.86 at 4 hpf; p = 0.0292, Student t-test for 1:1 ratio, n = 32, t = 1.07 at 8 hpf) (Fig 16).

### Repeatability

The raw image sequences for each of the colonies described above may be found in the Dryad Digital Repository (DOI:10.5061/dryad.5md80). Raw image sequences for replicate colonies at each of four ontogenetic stages have been also been deposited in the the same location, as have been image sequences corresponding to replicate day-long polyp behavior and multi-day stolon growth experiments.

### Equilibrial Dimensions

**Fig 17:**
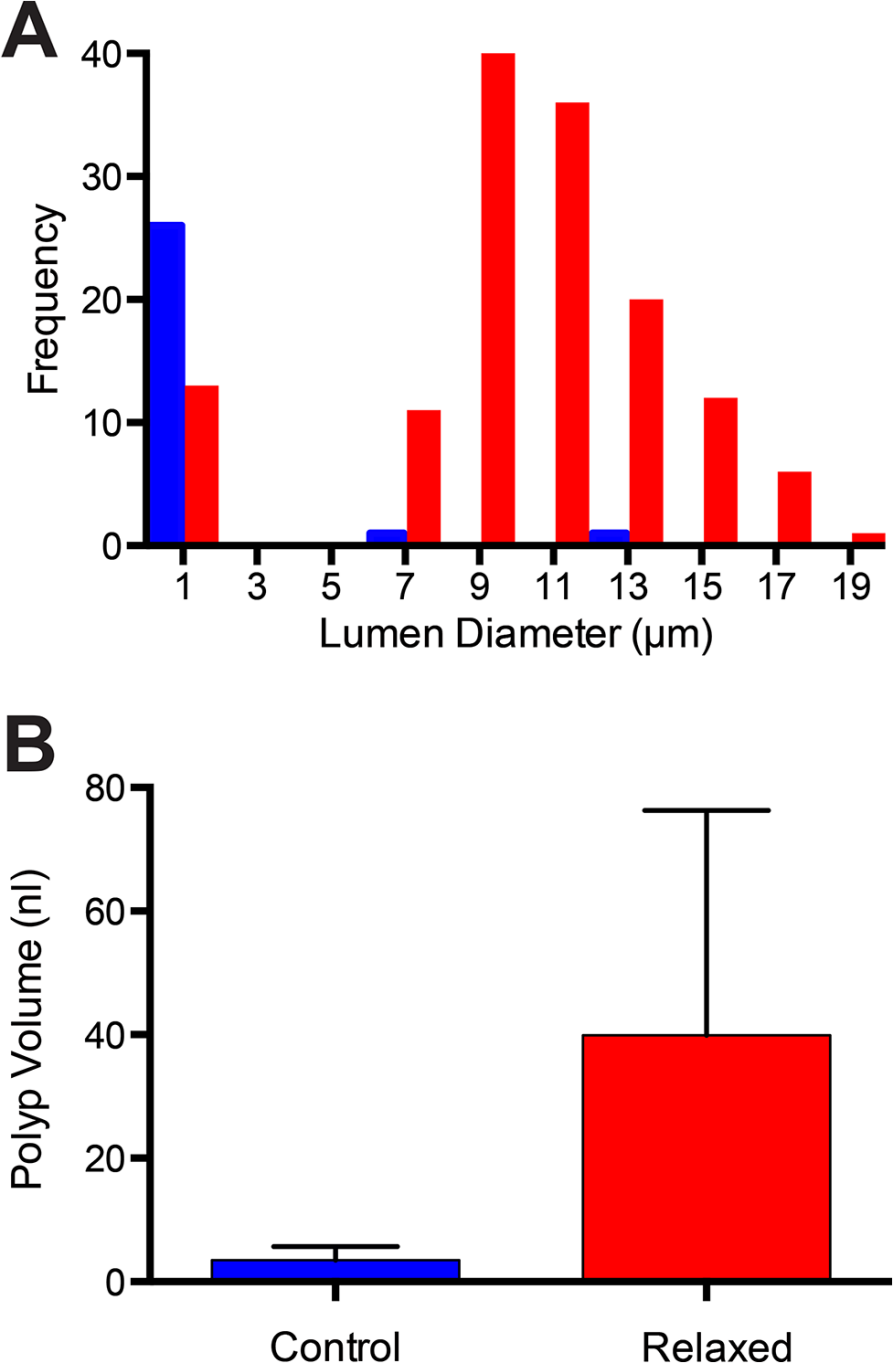
Equilibrial sizes. (A) Frequency distribution of equilibrial stolon lumen diameters. Peripheral tips, blue (n = 28); internal regions of stolons, red (n = 139). (B) Polyp volumes. Unrelaxed, blue (n = 11) and relaxed, red (n = 11). Polyps were of similar maximal length (700–800 μm).

Stolon tips display a markedly different rheology than that of older more internal regions of the hydrorhiza. When axial muscles are relaxed by application of blebbistatin virtually all stolon tips collapse, while the majority of internal regions retain an open lumen (Fig 17A). Under these same treatments, polyps balloon in size (Fig 17B), indicating that stolons are more resistant to changes in dimension than are polyps. In contrast to polyps, polyp buds behave as do peripheral stolon tips, with all buds observed collapsing (n = 13). Similarly all radial canals of medusae collapsed when relaxed (n = 32). The regions previously observed to exhibit enhanced luminance (stolon tips in Fig 9C, S5 Movie; polyp buds, Figs 4C and 4D; 9D; medusa, Fig 13, S6 Movie) share the feature that these regions collapse when pressure differentials within the colony are eliminated.

### Digestive Cell Distributions

**Fig 18:**
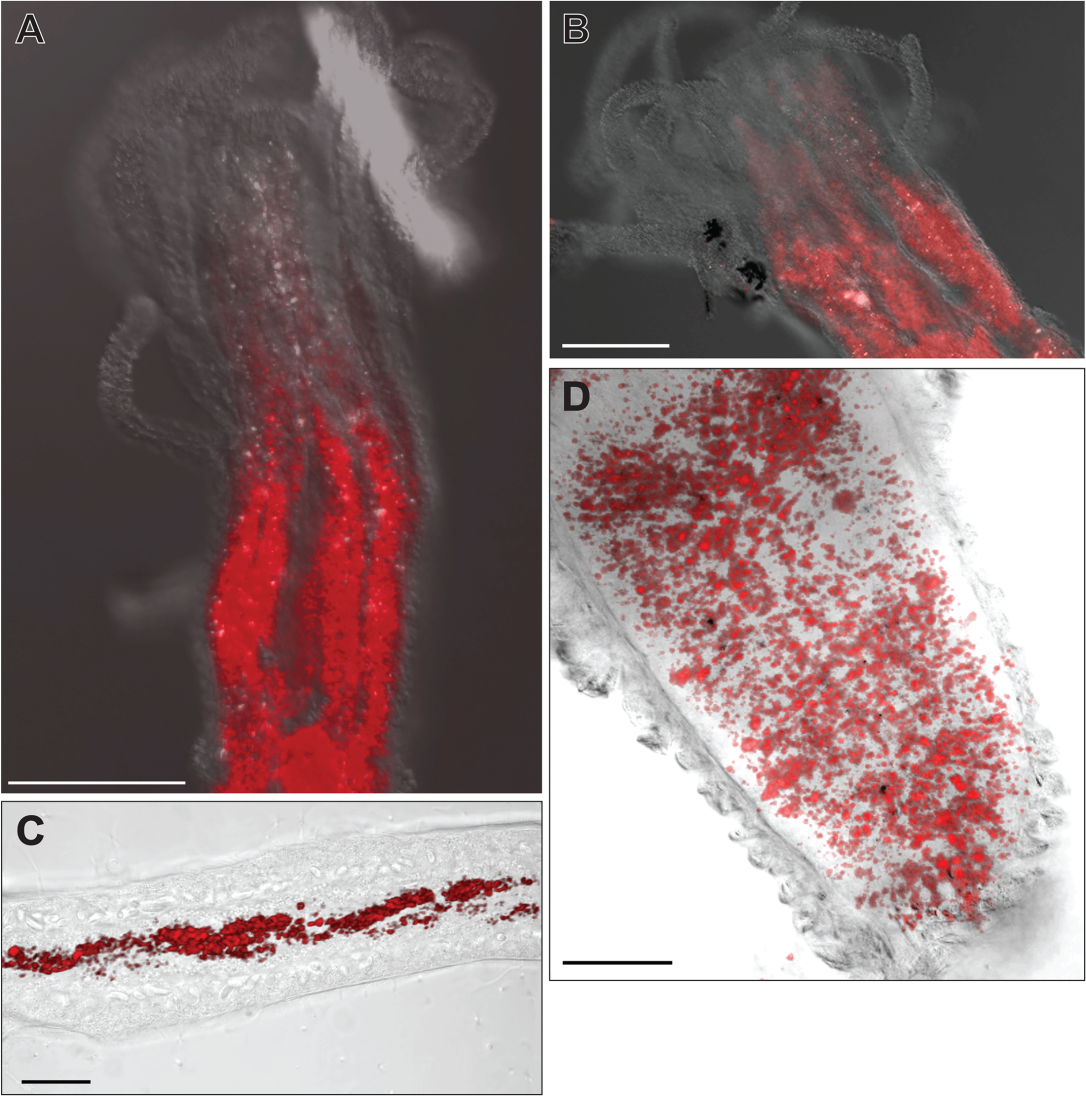
Distribution of label within digestive cells. (A) Feeding polyp. Projection of nine 6.5 μm optical sections. Scale bar = 100 μm. (B) Feeding polyp. Projection of ten 6.5 μm optical sections. Scale bar = 200 μm. (C) Stolon gastroderm. Scale bar = 20 μm. (D) Gastric cavity of reproductive polyp. Scale bar = 50 μm.

Our observations also revealed distinctive patterns in the spatial distribution of gastrodermal digestive cells (Fig 18, S2 Movie). In the feeding polyp digestive cells are arranged in longitudinal stripes along the oral-aboral axis running from the base of the hypostome to near the polyp-stolon junction (Fig 18A). At the hypostomal boundary these stripes are aligned with the positions of tentacle insertion (Fig 18B), hence are positioned between the hypostomal taeniolae. The gastric cavity of the medusae-bearing reproductive polyp lacks the radially symmetric intertaeniolar stripes of the feeding polyp (Fig 18D). Digestive cells in stolons are often, but not always, observed arranged in roughly helical axial strands (Fig 18C).

## Discussion

We have documented the distribution and absorption of fluorescent albumin at multiple stages of colony ontogeny at spatial scales ranging from that of digestive cells to entire colonies and at temporal scales from seconds to nearly a week. We find that food items are initially absorbed by the fed polyp and in regions immediately surrounding the fed polyp. This first phase of distribution and absorption was signaled by the dissolution of the food within hours post-ingestion and disappearance of both pellet fragments and solubilized label from the lumens of the gastrovascular system.

The spatial distribution of nutrient absorption in this phase approximated a diffusion pattern. This result may have been expected in that recent studies of the polyp-stolon junction show it to be a blind connection without morphologically distinct valves, nerves or musculature [16]. Thus, materials exiting the fed polyp into the vasculature would be expected to flow in accord with pressure differentials between the base of the polyp and positions within the hydrorhizal network. If the network displays equal connectivity in regions surrounding the fed polyp, these pressure differentials will be approximately equal and a diffusion pattern would be the expected outcome.

Following this first phase of distribution, label was found to re-enter the gastrovascular system in the form of particulates, which occasionally solubilize. This second phase of distribution peaks within the first day post-feeding, but continues for several days, resulting in distribution of the label to a broader region of the colony centered on the fed polyp and also consistent with that of a diffusive process (Fig 11B and 11C; S5 Movie).

Two considerations complicate the simple diffusion model of nutrient distribution. The hydrorhiza is a network of anastomosing canals and in growing colonies the connectivity of the network is unlikely to be equivalent in all directions from many polyps. Where connectivity is high, fluidic resistance may be expected to be lower and favor distribution in the direction of high connectivity. This effect may be responsible for the finding that nutrient absorption during the LD in large colonies prior to medusa production was greater toward the colony center than the colony periphery (S5 Fig).

A second consideration derives from the fact that different tissues display different rheologies. Hydroid intracellular digestion requires that a particulate come into contact with the gastrodermal cell surface and remain adjacent to that surface long enough period for microvilli or folds in the plasma membrane to surround it. This process may be more effective in those tissues with a more compliant rheology as they would be expected to locally collapse when pressure is low likely facilitating the absorption of food items. Three tissue compartments were found to collapse under the action of the non-muscle myosin II inhibitor blebbistatin: stolon tips, polyp buds, and radial canals of developing medusae (Fig 17A). Extreme distal portions of stolonal tips were found to be more luminous than more proximal regions of the same stolon (Fig 9C, S5 Movie), polyp buds were often found to be notably more luminous than adjacent hydrorhizal regions (Figs 4C and 12B), and medusae were more luminous than adjacent stolons or unfed polyps (Fig 14).

Thus nutrient absorption during both the early and the late distribution resembles a diffusion process, modulated by differences in tissue rheology and differences in hydrorhizal connectivity. In stark contrast to a diffusive process we observed that days after feeding some stolonal tips and polyp buds receive materials but not others. While the stolon tips that receive food are typically localized to a region of the colony that includes the fed polyp, it bears emphasis that some tips as close or closer to the fed polyp than others do not exhibit enhanced delivery (Figs 8,10; S5 Movie). This effect was observed both in the experiment where stolons were severed and later re-attached and in week-long observations of distribution and stolon growth. Thus, the during the late distribution a shift occurs from a interval within which distribution and absorption is diffusive to a later period when distribution and absorption are site-specific, directional, and clearly not approximated by diffusion.

These findings raise four unresolved larger issues to which we now turn.

### Cell Biology

A principal finding of this work is that fluorescent albumin was rapidly cleared from the gastrovascular system, only to reappear at later intervals as particulates that occasionally solubilize but that can remain in circulation for days after feeding (Figs 8–10, S3 and S4 Movies). The initial rapid absorption is consistent with earlier findings that digestion in cnidarians is primarily intracellular [22, 23]. While some proteolytic activity can be detected in the gastric cavity of a newly fed polyp [24], Lenhoff [25] showed that most protein is absorbed in unhydrolysed form and digested intracellularly. Lentz and Barnett [26] and Lentz [27] showed that digestive enzymes are associated not only with digestive vacuoles, as expected, but also with gastrodermal membranes.

Absorption of nutrients by the digestive cells occurs by both pinocytosis and phagocytosis. The phagocytic pathway allows for the absorption of particles of considerable size (> 5 μm) and may involve microvilli that adhere to the particle and subsequently fuse with one another to produce a continuous membrane. Alternatively one or more folds of the plasma membrane may encircle and engulf large particles [28–33]. These processes likely account for the rapid uptake of material in the fed polyp and for the absorption of particulates that become lodged in gastrovascular canals (Fig 5, S1 Movie).

What cell biological processes are responsible for the later release of tracer from the fed polyp into the hydroplasm? Two hypotheses merit attention. First, material may simply be ejected from the cell in much the same form as when it was first absorbed. Alternatively some form of apocrine secretion might be envisaged. Apocrine secretions may be conventional, where apical portions of the cell detach and enter the circulation. Alternatively, the cell might package the ingested material in specialized compartments and release these into circulation. McConnell [34] describes such a process in *Hydra*, where large ‘capsules’ containing food bodies entered the gastric cavity and ultimately burst and dispersed ‘food bodies’.

A candidate compartment is the discoidal coated vesicle (DCV). These organelles are known only from the digestive cells of hydroids and may well be diagnostic of the group [26, 27, 30–32, 35, 36]. The organelles densely populate the apical cytoplasm of digestive cells. When empty, DCV are flattened (600 Å), cigar-shaped (0.3–0.6 μm) structures with a distinctive internal surface. The latter marks them as distinct from typical pinocytotic vesicles. Slautterbach (1967) showed that they can open, fuse with the plasma membrane, absorb material from the gastric cavity, return to the cytoplasm and later dock with digestive vacuoles. The latter process can also be detected in Plate 63 of Lentz (1966). Both Lentz (1966) and Slautterbach (1967) show that ferritin fed to polyps becomes internalized within DCV. Slatterbach, however, found no localization of bovine serum albumin, ovoleithin, human haemoglobin, or horseradish peroxidase in DCV following injection of these materials into gastric cavities. Moreover, several other authors, studying the ultrastructure of absorption of *Artemia* nauplii, latex beads, and algal cells particles likewise failed to detect any uptake of these materials by DCV [30, 31, 37].

DCV are a hydroid curiosity and their function invites further study. We wonder whether their functions may include a route by which ingested materials might be compartmentalized and released as particulates to the gastrovascular system. This question may be readily explored by conventional transmission electron microscopy. The abrupt onset and sustained release of particulates in *Hydractinia* (Fig 7, S3 Movie) recommend the use of this organism for such an analysis.

### Radial Symmetry

The gastroderm of hydroid polyps displays a pronounced radially symmetric cellular organization [38–42]. At the hypostome, the gastroderm is strongly folded, with tentacles inserting between the folds. The folds are known as taeniolae, and the positions of tentacle insertion may be called intertaeniolar. Taeniolae are typically features of the oral region of the polyp; they diminish in size aborally. In some hydroids, notably including *Podocoryna* [40], tentiolae extend aborally to just above the base of polyp.

Our observations confirm that the radial organization of the feeding polyp gastroderm is not limited to the oral region of *Podocoryna* polyps but extends from the hypostome to the polyp base with digestive cells arranged in radially symmetric intertaeniolar bands (Fig 18A and 18B; S7 Movie). The pattern is reminiscent of the arrangement of digestive cells on the septa or mesenteries in other cnidarian groups, inviting the speculation that the arrangement of digestive cells observed here is a conserved radial feature diagnostic of the phylum. A comparative study of the arrangement of digestive cells across the phylum would be informative.

We did not observe oral-aboral stripes of digestive cells in the gastrodermal cavity of mature reproductive polyps (Fig 18B). Campbell [41], working with the *Hydractinia* observed that the oral blastostyle lacked taeniolae, consistent with the fact that these polyps lack tentacles and do not feed. *Podocoryna* differs from *Hydractinia* in this regard, in that *Podocoryna* reproductive polyps begin as tentacle-bearing, feeding polyps only to later lose their tentacles and begin to develop medusae. A study of the changes in radial organization of the gastroderm during the change in oral patterning of the *Podocoryna* sexual polyp would be of interest.

Of further interest is whether the radial organization of digestive cells in feeding polyps extends into and throughout the hydrorhiza. Our observations have not resolved this question. In some regions axial strands of digestive cells were found that appeared to wind helically along the proximal-distal axis of the stolon (Fig 18C), but this configuration was not everywhere evident. Further study is in order and would be facilitated by use of species with larger stolons and greater distances between polyps or uprights than is the case for *Podocoryna*. The techniques we used here to visualize digestive cells provide a ready and simple method for the surveys we suggest.

### Growth

A small literature has developed on colony pattern formation under varying nutritional conditions and our findings are germane to interpreting these findings. Encrusting hydroids grow by three processes: elongation of stolons, lateral branching of stolons, and budding of polyps (or, in some species, uprights bearing multiple polyps). Crowell [43] reared *Campanularia* colonies feeding them varying numbers of *Artemia* nauplii. His experiments revealed that peripheral stolon elongation and uprights/stolon length were little affected by starvation, while lateral branching was strongly suppressed. Braverman [44] showed the same effect using *Podocoryna*. These studies involved feeding entire colonies on a fixed feeding or starvation routine. Bumann and Buss [8] followed a different protocol, feeding polyps on one half of a *Podocoryna* colony, while starving the other half. They found that peripheral stolons on the unfed side elongated at the same rate as the fed side, but that lateral branching was strongly suppressed on the unfed side. The number of polyp buds/stolon length were comparable on the fed and unfed sides, but only polyps on the fed side grew beyond a certain minimum size. Thus, the fed side of colonies displayed a dense hydrorhizal network with numerous large polyps, while the unfed side of the colony was of comparable areal extent but was characterized by a sparse hydrorhiza and uniformly small polyps.

These findings are in broad accord with our studies of nutrient absorption. We found that absorption is localized in a diffusion pattern about the fed polyp, but that directional distribution to stolon tips and polyp buds also occurs. The close correspondence of colony form to patterns in nutrient absorption was unexpected. One might think that nutrients are delivered to particular sites, that those sites become mitotically active and enhanced tissue growth results. However, pattern formation in hydroids has long been appreciated to operate quite differently. Mitosis is often limited to specific regions and cells actively move along the mesoglea to generate changes in the sizes of tissue compartments [40, 45–54]. The fact that our studies of nutrient absorption and earlier studies of differential growth are in close accord would seem to necessarily imply that cell movements are preferentially directed to areas where nutrients have been absorbed.

### Site-specific Delivery

The directional delivery of nutrients to some stolons and not to others calls for an explanation. Polyps are known to be able to shut off delivery to the hydrorhizal system by constriction of circular muscles at the polyp base [14], but they lack specialized musculature to close off particular stolons that emanate from the polyp-hydrorhizal junction [16], which might otherwise render a finding of site-specific transport less puzzling.

Directional distribution and absorption in a fluid conducting system could be explained in three different ways. First, the variation in sites of delivery may reflect variability in the behavior of the pump that drives the fluid. This possibility is excluded by our observation that polyps were continuously oscillating with monotonically decreasing amplitude for a period of 28 hpf (S4 Fig). Whether polyp behavior is intermittent or erratic over longer periods of time is unknown. A second possibility is that material entered the gastrovascular fluid episodically and that these injections of labelled nutrient were absorbed in those stolons and polyp buds receiving flow at the time at which the nutrient was injected. We cannot fully exclude this possibility with the available evidence, but two lines of evidence argue against it. First, in all colonies we find a monotonic, rather than episodic, decline in luminosity of the fed polyp (Figs 3, 9, 11 and 13). Second, observation during late distribution in *H*. *symbiolongicarpus*, where a substantial portion of the colony was visualized, shows no obvious variability in the density of circulating material, despite the absorption of material at the colony periphery (S7 Movie).

The third and final possible explanation for directed delivery is that there is a mechanism to enhance volumetric flux from the fed polyp to particular polyp buds and stolon tips over others. This possibility is perhaps most likely given that the pump is continuously active and that the injection of material into the fluid is not markedly intermittent. Laminar flow in pipes is governed by Hagen-Poiseuille equation, which holds that volume flux scales with the 4^th^ power of radius [55]. Thus any mechanism that acts to increase the radius of stolon leading to a particular tip or bud will necessarily have a pronounced effect on the volume delivered to that location. A recent study has shown that stolons possess axial muscles which narrow the stolonal lumen when contracted [16]. This same study showed that nerves are present in the hydrorhiza at points just proximal to the stolon tip. When a stolon tip is severed, gastrovascular flow ceases immediately and only resumes when a new formed tip begins to pulsate [56–60]. These facts collectively suggest that stolon tips can trigger axial muscles in the stolon. This capacity may be expected to be shared by polyp buds, which at earliest stages are histologically identical to stolon tips [61]. If a subset of stolons and/or buds are triggering axial muscles, then volume flux will be disproportionate to those polyps and buds.

This mechanism requires that only some stolon tips are activating axial muscles at any given time. Wyttenbach [62] showed that in athecate hydroids only some stolon tips are actively pulsating at any given time. While the conditions under which stolons become active are unknown, our data are compatible with the suggestion that only actively pulsating stolons activate axial muscles to contract. In all hydroids, polyp buds and stolon tips only grow when they actively pulsate [21, 56–61, 63–67]. We have documented that a tip that received directed distribution of nutrients was actively elongating, while nearby stolons that did not receive nutrients were not (Fig 9). The episodic nature of the accumulation of nutrients in stolons in the severed stolon experiment (Fig 8) may be understood if different stolons become active at different times. Finally, the accumulation of the nutrients at the tips of those stolons receiving nutrients is compatible with our observations that the tip gastroderm collapses at lower pressures than more proximal regions of the stolon (Fig 17), allowing more time for membrane-associated absorptive processes to take place.

How then might a colony make a transition from a diffusive mode to a directional mode of nutrient distribution? Shortly after feeding, polyp contractions are generating large volume fluxes (S4 Fig), absorption is rapid (Figs 3, 9, 11 and 13) and advection generates a diffusion pattern (Figs 4A, 7A and 15). At times long after feeding, when polyp volume fluxes are much less (S4 Fig), we suggest only a fraction of the stolon tips and polyp buds remain active. Those tips and buds that remain active may be synchronized with one another, generating greater volume fluxes from the fed polyp to those tips and buds. The once diffusive delivery system becomes directional. This transition may underlie the observed change in the period of polyp oscillations late after feeding (S4 Fig). Indeed, a prior study of the dynamics of gastrovascular circulation in a one-polyp colony detected two dominant frequencies in the oscillatory dynamics [14], one of which was shown to match the period of the polyp oscillation. We suspect the second frequency, which became the dominant frequency late in the record, was derived from the hydrorhiza.

We note that the three possible explanations for directed distribution—irregularity in polyp pumping, episodic injection of material into the gastrovascular system, and tip-induced regulation of gastrovascular canal radius—are not mutually exclusive.

The transport system has adaptive consequences. During early and mid-ontogeny, the growing colony experiences both the diffusive and directional modes of nutrient distribution. Thus, the colony can both invest in growth in locations where recent success in capturing food occurred, and simultaneously expand at its periphery. During late ontogeny when peripheral tips are few or absent, reproductive polyps bear medusae. Each medusa is endowed with four radial canals, and each reproductive polyp bears multiple medusae. Hence reproductive polyps both have a large surface of area for absorption and a compliant rheology (Fig 17), effectively insuring that nutrients are directed disproportionally to reproduction. The gastrovascular system is an engineering marvel. The same components can not only sequentially direct materials locally and at a distance, but can similarly direct materials to growth and to reproduction at differing stages of colony ontogeny.

## Summary

1. An artificial food pellet containing Texas Red conjugated albumin is described. Solutes and pellet fragments are readily absorbed by hydroid digestive cells. No solid waste is generated and fed polyps do not regurgitate.
2. When a polyp is fed a single fluorescent pellet, the pellet is digested within the first 2–3 hpf, such that no fluorescent signal is detectable in the gastrovascular system. Rather, the label is found concentrated in digestive cells of the fed polyp and in a diffusion pattern in regions immediately surrounding the fed polyps.
3. After 6 hpf, label reappears within the gastrovascular system in the form of particulates that occasionally solubilize. This period of late distribution correlates with the loss of label from the fed polyp. The circulating material results in label reaching more distant regions of the colony, albeit in a broader diffusion pattern centered on the fed polyp.
4. Tracer molecules continue to circulate for over 6 days post-feeding. At later intervals, the distribution and absorption of label become concentrated in some, but not all, peripheral stolon tips and polyp buds. Distribution and absorption during this period shifts from diffusive to directional.
5. Unfed polyps and stolons accumulate comparable quantities of label. Polyp buds, stolon tips, and radial canals of developing medusae absorb relatively greater amounts of nutrient. Treatment of colony with the non-muscle myosin II inhibitor blebbistatin showed that these same tissues collapse when internal pressures within the colony are equalized.
6. Digestive cells within feeding polyps extend from the base of the hypostome to just above the polyp stolon junction in an intertaeniolar arrangement. Digestive cells are uniformly distributed in medusae-bearing polyps.

## Supporting Information

S1 Fig Orientation effects. (A, B) Two views of the same polyp. The food item (red) is imaged in the fluorescent channel and the polyp in DIC. Note that the polyp has rotated and the hypostome obscures part of the food pellet in (B). Scale bar: 200 μm.
(TIF)

S2 Fig Variability in relative luminance of the fed polyp in the inverted (green), side (red) and top up (magenta) orientations. Solid lines are 50 point moving averages. Large variations in the top up orientation are generated by the high density of medusa bearing polyps that often elect changes in the orientation of the fed polyp.
(TIF)

S3 Fig Mean luminance of selected time intervals for unfed polyps displaying nutrient absorption during the LD interval. The data is identical to that shown in Fig 3 except that the time scale has been expanded to provide greater detail. Color coding following that shown in Fig 1.
(TIF)

S4 Fig Mean luminance of a polyp imaged in the side orientation. (A) Full 28 hour record (n = 10,075). (B) 1.2–1.5 hpf.(C) 12.0–12.3 hpf. (D) 14.5–15.5 hpf. Note that polyp oscillates throughout the entire record, with the amplitude of oscillation declining with time. Further note that the period is much reduced at latest interval.
(TIF)

S5 Fig Distribution towards colony center in late ontogeny colony. (A) Superimposed on the colony schematic are 8 wedges each spanning 45°. Measurements were made in each wedge by outlining the stolons and calculating their mean luminance. Irregular areas defined in red were not measured. These regions correspond to locations where polyps obscured underlying stolons in part or all of the record. Color of wedges matched to time-series shown in (B,C). (B) Times-series for regions 45°–270° (northeast to west). (C) Time-series for regions 270–45° (northwest to northeast). Sampling interval for (B,C): 6 frames/minute, n = 3241.
(TIF)

S1 Movie Luminance in a portion of the hydrorhiza at 200X. Clip begins with a DIC image to capture the relevant network configuration followed by the fluorescent images. The clip repeats with the images color-coded for intensity. Time displayed as hours:minutes:seconds post-feeding. Images acquired at 6 frames per minute. Scale bar = 50 μm. The time-series for this record was shown in text Fig 6.
(MOV)

S2 Movie Luminance in a portion of the hydrorhiza at 100X. Clip begins with a DIC image to capture the relevant network configuration followed by the fluorescent images at 1.5, 10 and 12 hpf. Images acquired at 6 frames per minute. Scale bar = 50 μm. Selected images from this film were shown in text Fig 7.
(MOV)

S3 Movie Luminance in *Hydractinia symbiolongicarpus* at hourly intervals from 1–94 hpf. Scale bar = 2 mm.
(MOV)

S4 Movie Hydrorhizal luminance for the colony shown in Figs 10 and 11. First frame shows schematic of colony, followed by frames showing luminance on 6 consecutive days. Scale bar = 1 mm.
(MOV)

S5 Movie Tip Luminance and Growth. Portion of the colony shown in Figs 10 and 11 and S4 Movie, showing a growing stolon tip. First frame is schematic of region of interest with the fed polyp denoted by the red circle. Thereafter, luminance of selected region is shown at 3 hr intervals from 87–129 hpf, at which point the growing tip fuses with another stolon eliminating the tip. Not that the extreme distal end of the glowing tip is more luminous than proximal regions of the same or surrounding stolons throughout.
(MOV)

S6 Movie Distribution to a reproductive polyp bearing multiple medusae. 3–7 hpf. Scale bar: 250 μm.
(MOV)

S7 Movie Optical sections (0.5 μm) through two feeding polyps showing intertaeniolar distribution of digestive cells. Number of sections/polyp = 32. Scale bar: 200 μm.
(MOV)

S1 Software Code for calculating polyp volumes. (DOCX)

## References

[1] Boardman RS, Cheetam AH, Oliver WA Jr. Animal Colonies Stroudburg, Pennsylvannia: Dowden, Hutchinson & Ross; 1973.

[2] Larwood G, Rosen B. Biology and Systematics of Colonial Organisms London: Academic Press; 1979.

[3] Jackson JBC, Buss LW, Cook RE. Population Biology and Evolution of Clonal Organisms New Haven, Connecticut: Yale University Press; 1985.

[4] Buss LW. Growth by introsusception in hydractiniid hydroids In: Jackson JBC, Lidgard S, McKinney FK, editors. Evolutionary Patterns: Growth, Form and Tempo in the Fossil Record. Chicago: University of Chicago Press; 2001 p. 3–26.

[5] Dudgeon SR, Kübler JE. Hydrozoans and the shape of things to come. Advances in Marine Biology. 2011;59:107–44. 10.1016/B978-0-12-385536-7.00003-0 21724019

[6] Strehler BL, Crowell S. Studies on comparative physiology of aging. I. Function vs. age of *Campanularia flexuosa* . Gerontologia. 1961;5:1–8.

[7] Rees J, Davis LV, Lenhoff HM. Paths and rates of food distribution in the colonial hydroid *Pennaria* . Comparative Biochemistry and Physiology 1970;1970:309–16.

[8] Bumann D, Buss LW. Nutritional physiology and colony form in *Podocoryna carnea* (Cnidaria: Hydrozoa). Invertebrate Biology. 2008;127:368–80.

[9] Braverman M, Schrandt RG. Colony development of a polymorphic hydroid as a problem in pattern formation In: Rees WJ, editor. The Cnidaria and their Evolution. New York: Academic Press; 1966 p. 169–98.

[10] Blackstone NW, Buss LW. Shape variation in hydractiniid hydroids. Biological Bulletin. 1991;180:394–405. 29304663 10.2307/1542340

[11] Cadavid LF, Powell AE, Nicotra ML, Moreno M, Buss LW. An invertebrate histocompatibility complex. Genetics. 2004;167:357–65. 15166160 10.1534/genetics.167.1.357 PMC1470859

[12] Powell AE, Moreno M, Gloria-Soria A, Lakkis FG, Dellaporta SL, Buss LW. Genetic background and allorecognition phenotype in *Hydractinia symbiolongicarpus* . Genes, Genome, and Genomics. 2011;1:499–503. 10.1534/g3.111.001149 PMC3276163 22384360

[13] Powell AE, Nicotra ML, Moreno M, Lakkis FG, Dellaporta SL, Buss LW. Differential effect of allorecognition loci on phenotype in *Hydractinia symbiolongicarpus* (Cnidaria: Hydrozoa). Genetics. 2007;177:2101–7. 17947438 10.1534/genetics.107.075689 PMC2219508

[14] Dudgeon S, Wagner A, Vaisnys JR, Buss LW. Dynamics of gastrovascular circulation in the hydrozoan *Podocoryne carnea*: The 1-polyp case. Biological Bulletin. 1999;196:1–17. 25575381 10.2307/1543161

[15] Buss LW, Anderson C, Westerman E, Kritzberger C, Poudyal M, Moreno M, et al Allorecognition triggers autophagy and subsequent necrosis in the cnidarian *Hydractinia symbiolongicarpus* . Plos One. 2012;7:e48914 10.1371/journal.pone.0048914 23145018 PMC3493586

[16] Buss LW, Anderson C, Bolton EW. Muscular anatomy of the *Podocoryna carnea* hydrorhiza. Plos One. 2013;8, e72221 10.1371/journal.pone.0072221 23967288 PMC3743812

[17] Steinmetz PRH, Kraus JEM, Larroux C, Hammel JU, Amon-Hassenzahl A, Houliston E, et al Independent evolution of striated muscles in cnidarians and bilaterians. Nature. 2012;487:231–4. 10.1038/nature11180 22763458 PMC3398149

[18] Kolega J. Phototoxcity and photinactivation of blebbistatin in UV and visible light. Biochem Biophys Res Commun. 2004;320:1020–5. 15240150 10.1016/j.bbrc.2004.06.045

[19] Cartwright P, Buss LW. Expression of a *Gsx* parahox gene, *Cnox-2*, in colony ontogeny in *Hydractinia* (Cnidaria: Hydrozoa). Journal of Experimental Zoology. 2006;306B:1–10. 10.1002/jez.b.21106 16615106

[20] Overton J. Intercellular connections in outgrowing stolon of *Cordylophora* . J Cell Biol. 1963;17:661–71. 19866630 10.1083/jcb.17.3.661 PMC2106220

[21] Hale LJ. Cell movements, cell division and growth in the hydroid *Clytia johnstoni* . J Embryol Exp Morph. 1964;12:517–38. 14207037

[22] Metschnikoff E. Uber die intracelluläre Verdauung bei Coelenteratan. Zool Anz. 1880;3:261–3.

[23] Parker J. On the histology of *Hydra fusca* . Proceedings of the Royal Society of London. 1879;30:61–6.

[24] Beutler R. Experimentelle Untersuchungen über die Verdauung bei *Hydra* . Journal of Comparative Physiology. 1924;1:1–56.

[25] Lenhoff HM. Digestion of protein in *Hydra* as studied using radioautography and fractionation by differential solubilities. Experimental Cell Research. 1961;23:335–53. 13760830 10.1016/0014-4827(61)90043-x

[26] Lentz TL, Barrnett RJ. Surface specializations of *Hydra* cells: the effect of enyzme inhibitors on ferritin uptake. Journal of Ultrastructural Research. 1965;13:192–211. 10.1016/s0022-5320(65)80096-x 4378805

[27] Lentz TL. The Cell Biology of *Hydra* Amsterdam: North-Holland Publishing Company; 1966.

[28] Gautheir GF. Cytological studies on the gastroderm of *Hydra* . Journal of Experimental Zoology. 1963;152:13–40.

[29] Lunger PD. Fine-structural aspects of digestion in a colonial hydroid. Journal of Ultrastructural Research. 1963;9:362–80.

[30] Afzelius BA, Rosén B. Nutritive phagocytosis in animal cells. An electron microscopic study of the gastroderm of the hydroid *Clava squamata* Müll. Zeitschrift für Zellforschung. 1965;67:24–33. 4379911

[31] Cook CB, D'Elia CF, Muscatine L. Endocytic mechanisms of the digestive cells of *Hydra viridis* . Cytobios. 1979;89:17–31. 755609

[32] McNeil PL. Mechanisms of nutritive endocytosis. I. Phagocytic versatility and cellular recognition in *Chlorohydra* digestive cells, a scanning electron microscopic study J Cell Sci. 1981;49:311–39. 7309809 10.1242/jcs.49.1.311

[33] McNeil PL. Mechanisms of nutritive endocytosis. III. A freeze-fracture study of phagocytosis by digestive cells of *Chlorohydra* . Tissue & cell. 1984;16:519–33. 6484935 10.1016/0040-8166(84)90028-4

[34] McConnell CH. A detailed study of the endoderm of *Hydra* . Journal of Morphology and Physiology. 1931;52:249–75.

[35] Slautterback DB. Coated vesicles in absorptive cells of *Hydra* . J Cell Sci. 1967;2:563–72. 4384036 10.1242/jcs.2.4.563

[36] Hündgen M. The biology of colonial hydroids. I. The morphology of the polyp of *Eirene viridula* (Thecata: Campanulinidae). Marine Biology. 1978;45:79–92.

[37] McNeil PL. Mechanisms of nutritive endocytosis. I. Phagocytic versatility and cellular recognition in *Chlorohydra* digestive cells, a scanning electron microscopic study. J Cell Sci. 1981;49:311–39. 7309809 10.1242/jcs.49.1.311

[38] Hamann O. Der Organismus der Hydroidpolypen. Jena Z Naturiss. 1882;15:473–544.

[39] Kühn A. Entwicklungsgeschichte und Verwandschaftsbeziehungen der Hydrozoen. Ergebn Fortschr Zool. 1914;4:1–284.

[40] Braverman M. Studies on hydroid differentiation. III. The replacement of hypostomal gland cells of *Podocoryna carnea* . Journal of Morphology. 1968;126:95–106. 4386939 10.1002/jmor.1051260106

[41] Campbell RD. Cell proliferation and morphological patterns in the hydroids *Tubularia* and *Hydractinia* . J Embryol Exp Morph. 1967;17:607–17. 4383059

[42] Mokady O, Dick MD, Lackschewitz D, Schierwater B, Buss LW. Over one-half billion years of head conservation? Expression of an ems class gene in *Hydractinia symbiolongicarpus* (Cnidaria: Hydrozoa). Proceedings of the National Academy of Sciences, USA. 1998;95:3673–8. 10.1073/pnas.95.7.3673 PMC19894 9520424

[43] Crowell S. Differential responses of growth zones to nutritive level, age, and temperature in the colonial hydroid *Campanularia* . Journal of Experimental Zoology. 1957;134:63–90. 13428946 10.1002/jez.1401340104

[44] Braverman M. The cellular basis of morphogenesis and morphostasis in hydroids. Annual Review of Oceanography and Marine Biology. 1974;12:129–221.

[45] Tripp K. Die Regenerationsfähigkeit von Hydren in den verschiedenen Körperregionen Nach Regulations und Transplantationsversuchen. Z Wiss Zool. 1928;132:476–525.

[46] Brien P, Reniers-Decoen M. La croissance, la blastogénèse, l'ovogénèse chez Hydra fusca (Pallas). Bull Biol France Belgique. 1949;83:293–336.

[47] Brien P. Contribution á l'ètude des hydres d'eau douce (*Hydra fusca*, *H*. *viridis*, *H attenuata*) Croissance et Reproduction. Bull Biol France Belgique. 1951;76(277–296).

[48] Burnett AL. The growth process in *Hydra* . Journal of Experimental Zoology. 1961;146:21–83.

[49] Campbell RD. Tissue dynamics of steady state growth in *Hydra littoralis*.I. Patterns of cell division. Developmental Biology. 1967;15:487–502. . 4382248 10.1016/0012-1606(67)90039-5

[50] Campbell RD. Tissue dynamics of steady state growth in *Hydra littoralis* .2. Patterns of tissue movement. Journal of Morphology. 1967;121:19–28. . 4166265 10.1002/jmor.1051210103

[51] Campbell RD. Tissue dynamics of steady state growth in *Hydra Littoralis*. 3. Behavior of specific cell types during tissue movements. Journal of Experimental Zoology. 1967;164:379–91. .

[52] Shostak S. Bud movements in *Hydra* . Science. 1967;155:1567–8. 17830051 10.1126/science.155.3769.1567

[53] Shostak S, Kankel D. Morphogenetic movements during budding in *Hydra* . Developmental Biology. 1967;15:451–63. 4382247 10.1016/0012-1606(67)90037-1

[54] Braverman M. Studies on hydroid differentiation. VII. The hydrozoan stolon. Journal of Morphology. 1971;135:131–52. 4400260 10.1002/jmor.1051350202

[55] Bird RB, Stewart WE, Lightfoot EN. Transport Phenomena New York: John Wiley & Sons; 2002.

[56] Hale LJ. Contractility and hydroplasmic movements in the hydroid *Clytia johnstoni* . Q J Microsc Sci. 1960;101:39–51.

[57] Wyttenbach CR. The dynamics of stolon elongation in the hydroid, *Campanularia flexuosa* . Journal of Experimental Zoology. 1968;167:333–51.

[58] Wyttenbach CR. The role of hydroplasmic pressure in stolonic growth movements in the hydroid, *Bougainvillia* . Journal of Experimental Zoology. 1973;186:79–90.

[59] Wyttenbach CR. Cell movements associated with terminal growth in colonial hydroids. American Zoologist. 1974;14:699–717.

[60] Donaldson S. Terminal motility in elongating stolons of *Proboscidactyla flavicirrata* . American Zoologist. 1974;14:735–44.

[61] Beloussov LV. Growth and morphogenesis of some marine Hydrozoa according to histological data and time-lapse studies. Publications of the Seto Marine Laboratory. 1973;20:315–36.

[62] Wyttenbach CR, Crowell S, Suddith RL. Variation in the mode of stolon growth among different genera of colonial hydroids, and their evolutionary implications. Journal of Morphology. 1973;139:363–76. 30352499 10.1002/jmor.1051390306

[63] Berrill NJ. The polymorphic transformations of *Obelia* . Q J Microsc Sci. 1949;90:235–64.

[64] Berrill NJ. Growth and form in gymnoblastic hydroids.1. Polymorphic development in *Bougainvillia* and *Aselomaris* . Journal of Morphology. 1949;84:1–30. 18108870 10.1002/jmor.1050840102

[65] Beloussov LV, Badenko LA, Kurilo LF, Katchuri Al. Cell movements in morphogenesis of hydroid polypes. J Embryol Exp Morph. 1972;27:317–37. 4402664

[66] Beloussov LV, Labas JA, Kazakova NI, Zaraisky AG. Cytophysiology of growth pulsations in hydroid polyps. Journal of Experimental Zoology. 1989;249:258–70.

[67] Kosevitch IA. Mechanics of growth pulsations as the basis of growth and morphogenesis in colonial hydroids. Russian Journal of Developmental Biology. 2006;37:90–101. 16634200

