# Supplementary figures and images for: Nutrient Distribution and Absorption in the Colonial Hydroid Podocoryna carnea Is Sequentially Diffusive and Directional
Source: PLoS One. 2015 Sep 11;10(9):e0136814. doi: 10.1371/journal.pone.0136814 (PMC4567339; doi:10.1371/journal.pone.0136814)

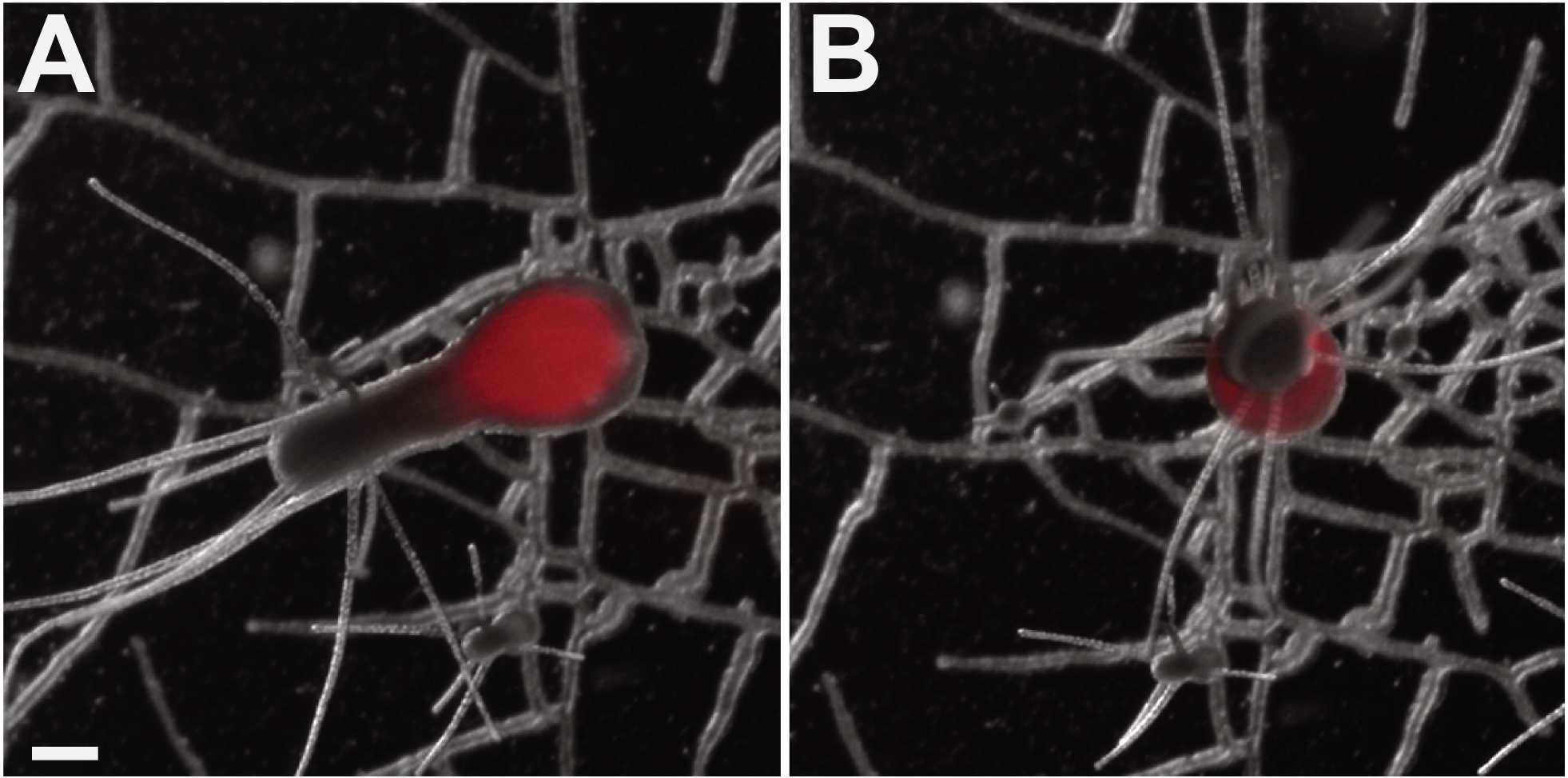

Supplement: S1 Fig — (A, B) Two views of the same polyp. The food item (red) is imaged in the fluorescent channel and the polyp in DIC. Note that the polyp has rotated and the hypostome obscures part of the food pellet in (B). Scale bar: 200 μm. (TIF) [file pone.0136814.s001.tif]

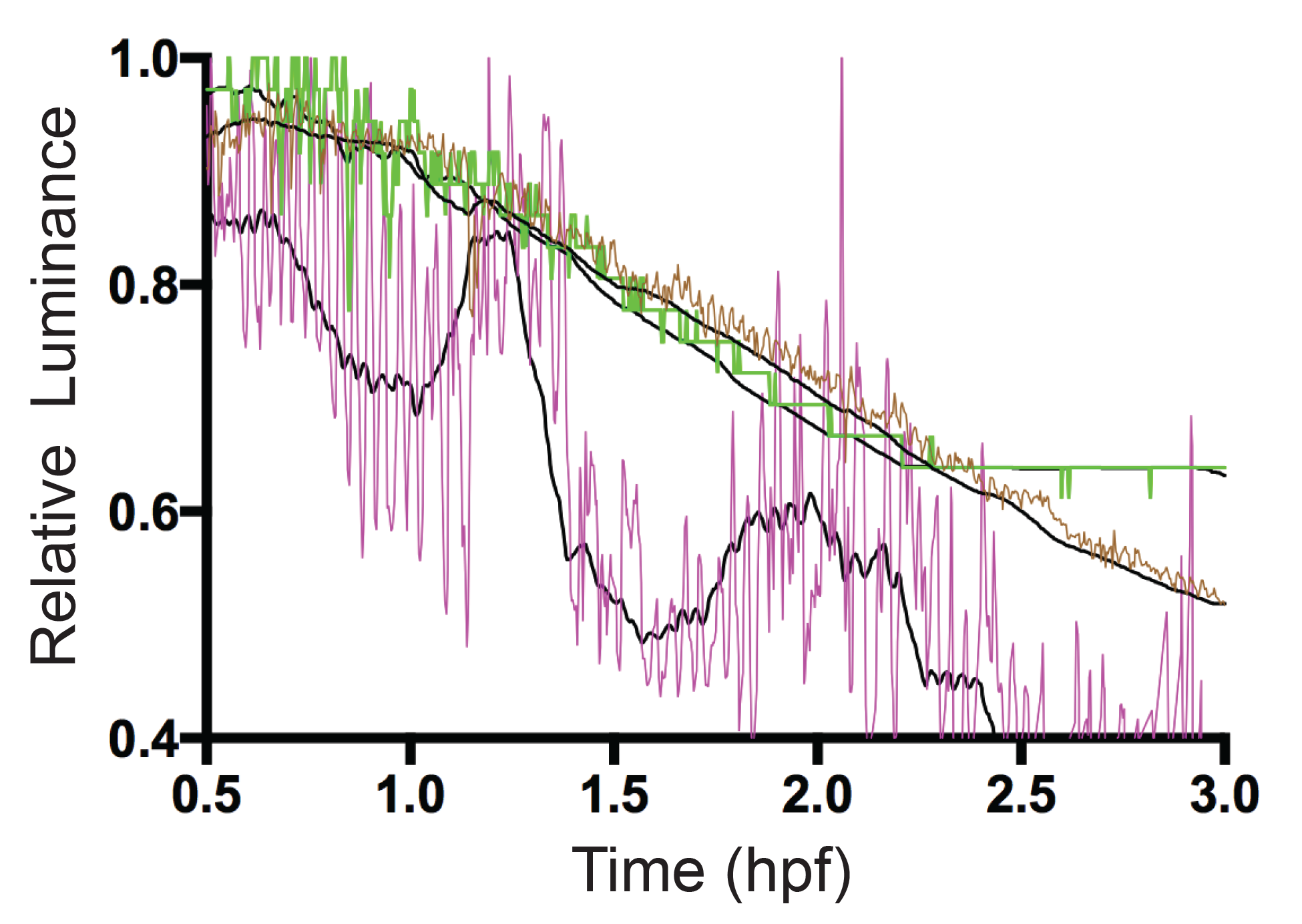

Supplement: S2 Fig — Solid lines are 50 point moving averages. Large variations in the top up orientation are generated by the high density of medusa bearing polyps that often elect changes in the orientation of the fed polyp. (TIF) [file pone.0136814.s002.tif]

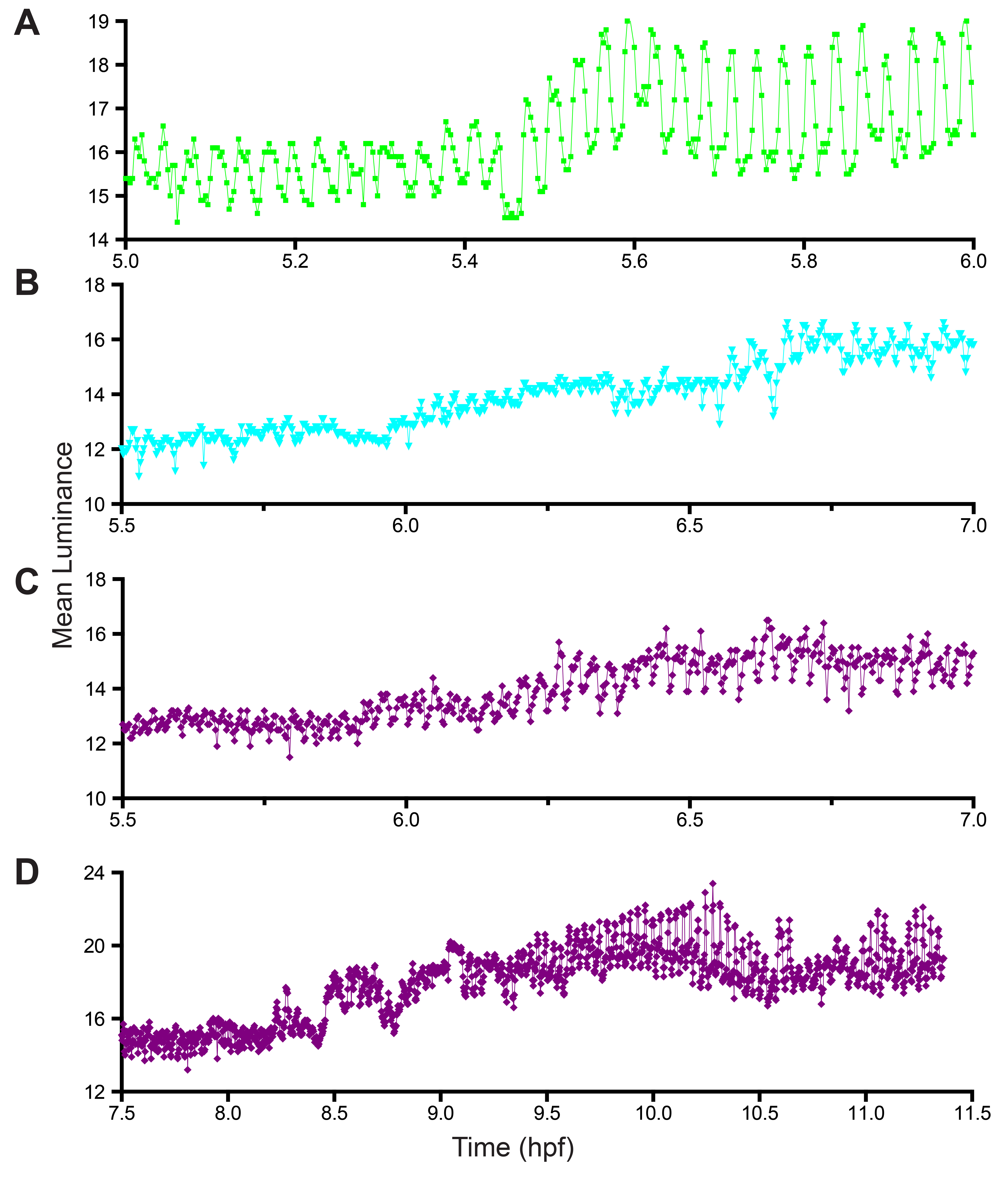

Supplement: S3 Fig — The data is identical to that shown in Fig 3 except that the time scale has been expanded to provide greater detail. Color coding following that shown in Fig 1. (TIF) [file pone.0136814.s003.tif]

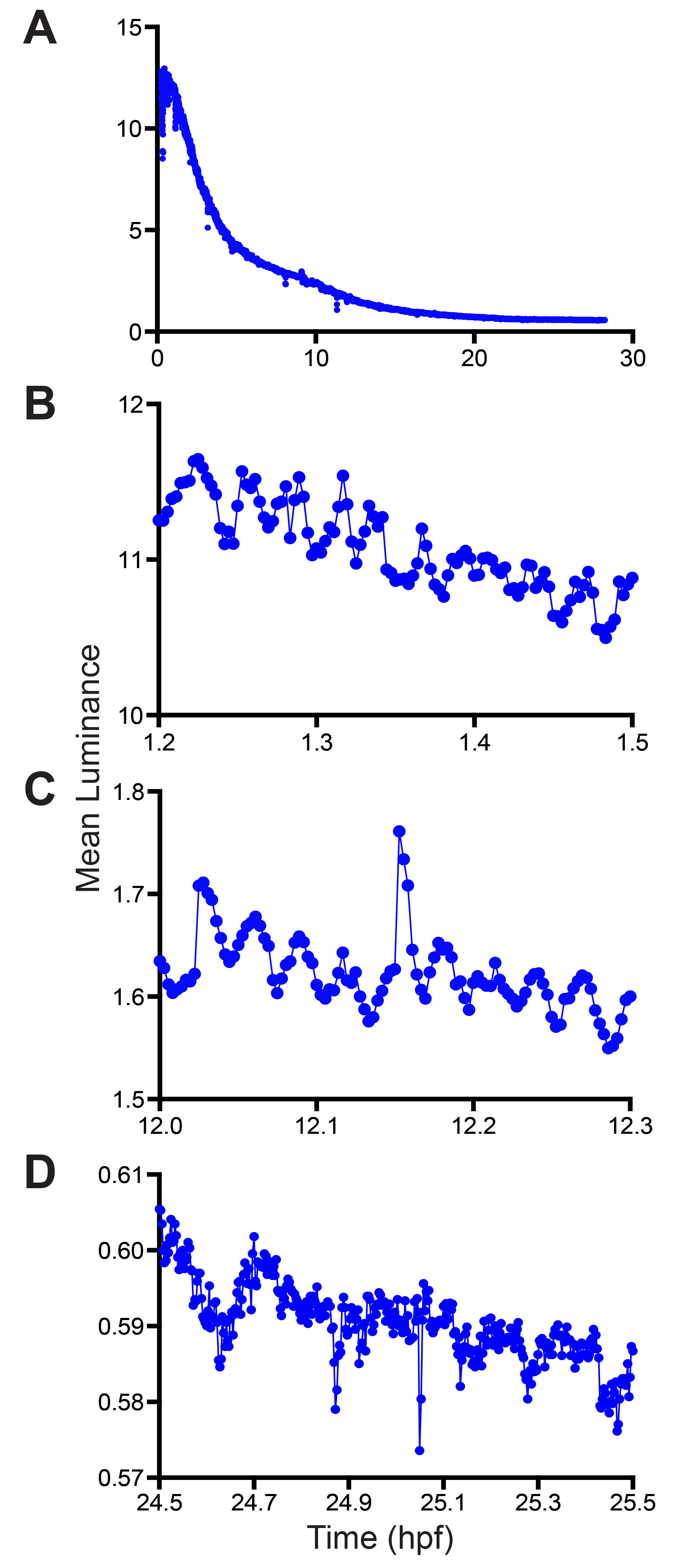

Supplement: S4 Fig — (A) Full 28 hour record (n = 10,075). (B) 1.2–1.5 hpf.(C) 12.0–12.3 hpf. (D) 14.5–15.5 hpf. Note that polyp oscillates throughout the entire record, with the amplitude of oscillation declining with time. Further note that the period is much reduced at latest interval. (TIF) [file pone.0136814.s004.tif]

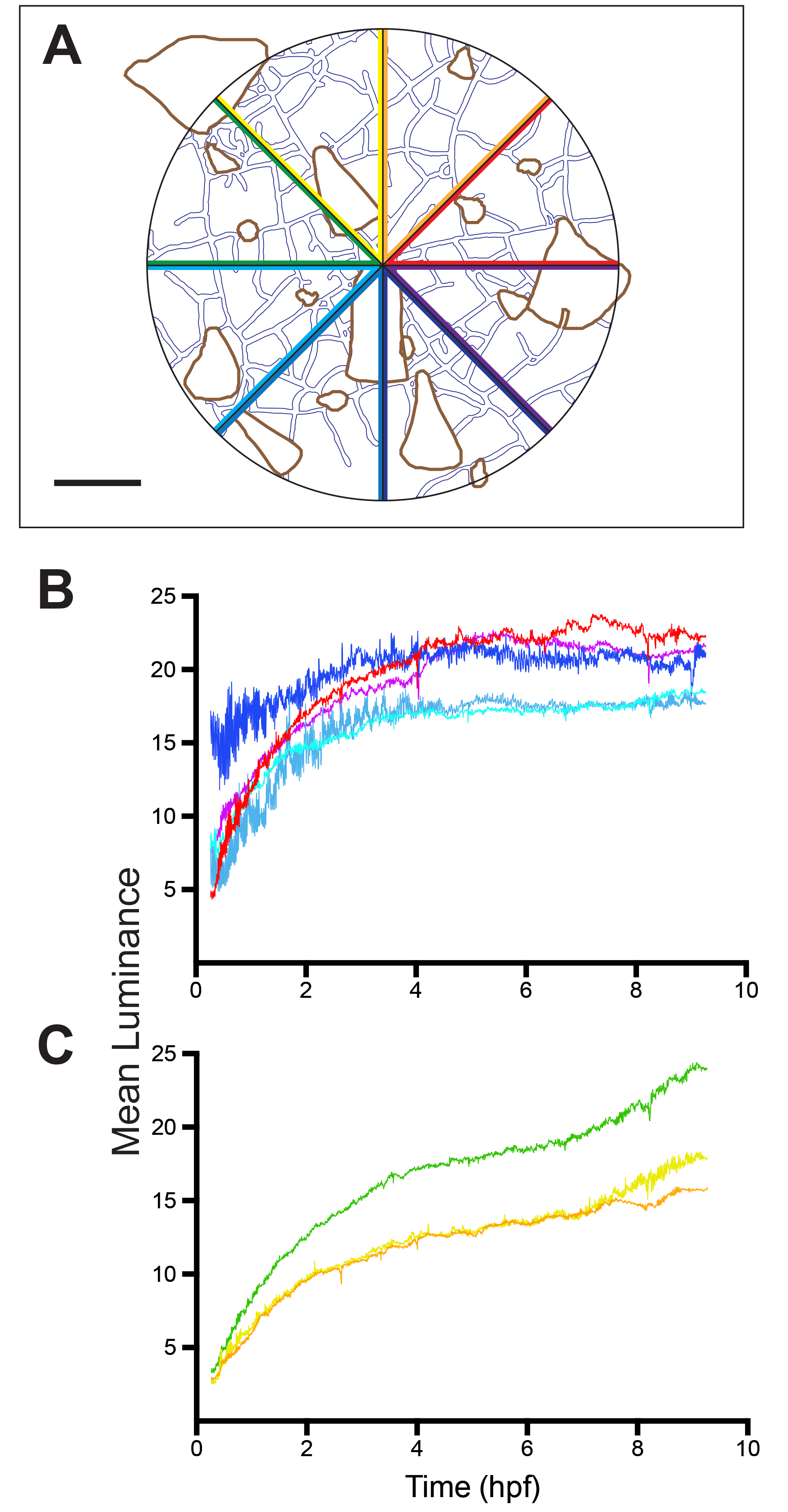

Supplement: S5 Fig — (A) Superimposed on the colony schematic are 8 wedges each spanning 45°. Measurements were made in each wedge by outlining the stolons and calculating their mean luminance. Irregular areas defined in red were not measured. These regions correspond to locations where polyps obscured underlying stolons in part or all of the record. Color of wedges matched to time-series shown in (B,C). (B) Times-series for regions 45°–270° (northeast to west). (C) Time-series for regions 270–45° (northwest to northeast). Sampling interval for (B,C): 6 frames/minute, n = 3241. (TIF) [file pone.0136814.s005.tif]
