# Supplementary material for: Nutrient Distribution and Absorption in the Colonial Hydroid Podocoryna carnea Is Sequentially Diffusive and Directional
Source: PLoS One. 2015 Sep 11;10(9):e0136814. doi: 10.1371/journal.pone.0136814 (PMC4567339; doi:10.1371/journal.pone.0136814)
Supplement: S1 Software — (DOCX) [file pone.0136814.s013.docx]

**S1 Software**

# This R code reads csv files with

# rows that contain, first the polyp length in microns,

# followed by the segment diameter 7 locations up

# from the base (also in microns).

# columns: one for each polyp in the dataset

# use:

# setwd('***')

# to go to the directory containing the csv file

#----------Choose as relevant

bloat1=read.csv(file="Bleb-pol-bloat.csv")

dataset="Bleb-pol-bloat"

simsize=read.csv(file="Bleb-pol-sim-size-data.csv")

dataset="Bleb-pol-sim-size-data"

dim1=dim(bloat1)

dim2=dim(simsize)

allrawdat=cbind(bloat1,simsize)

dataset="bloat and simsize"

dimraw=dim(allrawdat)

#[1] 8 22

matplot(allrawdat[(2:dimraw[1]),],type='l')

#------------

numpol=dimraw[2] # number of polyps

nummeas=dimraw[1]-1

volvec=c(numpol) # to dimension an array

Lvec=c(numpol) # to dimension an array

diammat=matrix(0,(nummeas+2),numpol) # to dimension an array

zlocmat=matrix(0,(nummeas+2),numpol) # to dimension an array

for(ip in 1:numpol){

L=allrawdat[1,ip]

Lvec[ip]=L # polyp length

measures=allrawdat[2:(nummeas+1),ip] # diameters at various z locations

dlist=measures

auglist=c(0,dlist,0)

# the diameters at the top and bottom of the polyps are assumed to be zero

# these zeros are included in an augmented diameter array.

diammat[,ip]=auglist

dz=L/(length(auglist)-1) # the distance between diameter measurements

# zlocmat contains the z location up from the polyp base

zlocmat[,ip]=c(0,dz,2*dz,3*dz,4*dz,5*dz,6*dz,7*dz,L)

last=length(dlist)+1

last

dvol=c(1:last)*0

for(i in 1:(last)){

h=dz

r1= auglist[i]/2 # convert diameters to radii

r2= auglist[i+1]/2

#formula to calculate incremental volumes of a right circular cone

dvol[i]=(pi/3)*h*(r1*r1+r2*r2+r1*r2)

zlocmat[i,ip]=(i-1)*dz

}

vol=sum(dvol) # total polyp volume in cubic microns

volnL=vol*10^(-6) # convert cubic microns to nanoliters

print(ip)

print(volnL)

volvec[ip]=volnL

zlocmat[(last+1),ip]=L

}

par(mfrow=c(1,1)) # various plots for visualization

plot(Lvec,volvec,ylim=c(0,max(volvec)))

matplot(zlocmat[1:(nummeas+2),],diammat[(1:(nummeas+2)),],type='l',

col='black')

matplot(zlocmat[2:(nummeas+1),],diammat[(2:(nummeas+1)),],type='l',

col='black')
